# Hypothalamus Amyloid Levels Are Associated with Early Sex-Dependent Alterations in Peripheral Energy Homeostasis in TgF344-AD Rats

**DOI:** 10.1007/s12035-026-06014-4

**Published:** 2026-07-02

**Authors:** Caleb M. Levine, Cameron Caggiano, Thea Anderson, Michael A. Kelberman, David Weinshenker, Hannah L. Lail, Desiree Wanders, Debra A. Bangasser, Scott E. Kanoski, Marise B. Parent

**Affiliations:** 1 Neuroscience Institute, Petit Science Center, Georgia State University, PO Box 5030, Atlanta, GA 30302-5030 USA; 2 Department of Human Genetics, Emory School of Medicine, Whitehead Biomedical Research Building, 615 Michael St, Suite 301, Atlanta, GA 30322 USA; 3 Department of Nutrition, Georgia State University, Urban Life Building, PO Box 4019, Atlanta, GA 30302-4019 USA; 4 Center for Behavioral Neuroscience, Petit Science Center, Georgia State University, PO Box 5030, Atlanta, GA 30302-5030 USA; 5 Human and Evolutionary Biology Section, Department of Biological Sciences, Dornsife College of Letters, Arts and Sciences, University of Southern California, 3616 Trousdale Parkway, AHF-252, Los Angeles, CA 90089-0372 USA; 6 Department of Psychology, Georgia State University, Urban Life Building, 140 Decatur St, Atlanta, GA 30303-3083 USA

**Keywords:** Alzheimer’s disease, Blood glucose, Metabolism, Brown adipose tissue, Uncoupling 1 protein

## Abstract

We reported previously that diet-induced obesity exacerbates early-stage Alzheimer’s disease (AD)–like pathology in TgF344-AD rats. Those findings also suggested that they may be prone to weight gain during early AD development, raising the possibility of early hypothalamic dysfunction. In this study, we investigate this by determining whether hypothalamic soluble amyloid-β_40_ (Aβ_40_) and amyloid-β_42_ (Aβ_42_) are detectable during early AD development in TgF344-AD rats and whether their presence is associated with alterations in energy homeostasis. Energy intake, body composition, whole body glucose clearance, body temperature rhythms, interscapular brown adipose tissue (iBAT) mass, and iBAT expression of the thermogenic uncoupling protein 1 (UCP1) were assessed. The results demonstrate that Aβ_40_ and Aβ_42_ are detectable in the hypothalamus during the early stages of AD development and that hypothalamic but not cortical Aβ_42_ is associated with impaired glucose regulation in males and iBAT mass in females. Male TgF344-AD rats became heavier than wild-type (WT) littermates by 5 weeks of age, with a similar increase emerging later in females (~ 5 months). Female TgF344-AD rats consumed more chow and a high-fat high-sugar (HFHS) diet, gained more weight on that diet, and showed reduced UCP1, effects not seen in males. Both sexes exhibited elevated body temperatures. This increase was restricted to the dark phase in females, coinciding with excess caloric intake. Finally, HFHS feeding impaired glucose regulation in male but not female TgF344-AD rats. These findings suggest hypothalamic Aβ_42_ may contribute to sex-specific disruptions in peripheral energy homeostasis that may be an early symptom of AD development.

**Supplementary Information:**

The online version contains supplementary material available at 10.1007/s12035-026-06014-4.

## Introduction

Alzheimer’s disease (AD) is the most common form of dementia, affecting an estimated 416 million people worldwide, and AD prevalence is expected to continue to increase [1, 2]. Currently, the most effective treatments available only modestly attenuate AD symptoms and slow the rate of decline, and neither curative nor preventative solutions are available [3, 4]. AD is characterized by the progressive deposition of amyloid-β peptides (Aβ) as plaques, the development of neurofibrillary tangles (NFTs) composed primarily of the hyperphosphorylated microtubule-associated protein tau (ptau), and eventual neuronal death [5, 6]. AD starts to develop during middle age decades before dementia is diagnosed and progresses in stages. The preclinical stage is the earliest asymptomatic stage where there is pathology, but no perceptible cognitive decline and noncognitive symptoms may be present [7]. This pathology includes elevated Aβ and changes in tau that often go undetected until mild cognitive impairment (MCI) appears during the prodromal stage, which in turn lasts until frank dementia emerges during the clinical stage [8–13].

Many risk factors associated with AD exert their effects during the preclinical and prodromal periods (i.e., during middle age). Of note, midlife obesity doubles AD risk and predicts earlier AD onset and more severe pathology [14–16]. This is a significant concern because the worldwide prevalence of obesity has nearly tripled since 1975 [17]. Although midlife obesity is associated with increased AD risk [18–23], late-life obesity is not, suggesting that the risk interactions between obesity and AD occur during preclinical/prodromal AD stages. Even though revised guidelines have allowed the diagnosis of AD at earlier stages of pathology based on positive imaging or blood biomarkers, not all will go on to develop symptoms of AD [24]. As a result, it remains very challenging to investigate the mechanisms underlying risk factors during preclinical and prodromal AD.

Given these challenges, nonhuman animal models are vital for investigating the neural changes that occur during early AD development and for understanding how risk factors increase the likelihood of transitioning from early to later stages. Of all rodent models currently available, the TgF344-AD rat, which expresses the Swedish mutant human amyloid precursor protein (APPsw) and Δexon9 mutant human presenilin-1 (PS1 ΔE9), appears to most closely recapitulate human AD trajectory [25]. TgF344-AD rats develop elevations in Aβ oligomers (Aβos), intraneuronal Aβ, plaques, progressive memory deficits, neuroinflammation, gliosis, and apoptotic neuronal injury/loss in an age-dependent manner [25, 26]. Unique to this model, Aβ triggers endogenous tauopathy consisting of NFT-like aggregated rat ptau in brain areas relevant to early AD pathology, such as the locus coeruleus, hippocampus, cortex, and entorhinal cortex [25–30]. This is a significant advantage of this model because tau pathology is more closely related to the etiology of neurodegeneration and dementia than Aβ [31, 32].

Importantly, pathology proceeds through characteristic preclinical, prodromal, and “clinical” stages as TgF344‐AD rats age. The first signs of the preclinical period appear in TgF344-AD between 4 and 6 months of age with increased anxiety [33], sleep disturbances [34], and spatial orientation defects [35]. Spatial memory defects occur inconsistently at 6 months [33, 36], and prodromal symptomology becomes clearer by 7 months with more spatial memory [36–38], cognitive flexibility [39], and working memory [40] reductions. By 10–12 months, TgF344-AD rats have impaired place avoidance learning [41], continued spatial memory defects [35, 42], and disrupted novel object performance [43]. Beyond 14 months, TgF344-AD rats are in their clinical phase and exhibit substantial cognitive impairment [35, 44–47], aggregated ptau [35, 48], and neuronal loss [35].

Evidence suggests that peripheral energy homeostasis may be disrupted in TgF344-AD rats. For example, TgF344-AD rats fed standard chow weigh more than their WT counterparts [49–51] and have elevated blood lipid levels [51]. Moreover, feeding high-fat/high-sugar (HFHS) diets to TgF344-AD rats, particularly females, produces larger elevations in weight gain and adiposity than in WT rats [52, 53]. The findings from the last two studies are particularly intriguing as they examined the effects of diet-induced weight gain during the prodromal period in these rats, when obesity is most impactful as a risk factor for future AD development in humans. This evidence of disrupted energy homeostasis during early AD development raises the possibility that the hypothalamus, which is critical for energy homeostasis [54, 55], is impaired during early AD development in these rats. In support, Aβ plaques and NFTs have been observed in the hypothalamus of patients with AD, although most of this pathology has been identified in later stages of AD development [56]. Importantly, a recent systematic review demonstrated that patients with mild to moderate AD have reduced hypothalamic volume, suggesting that the hypothalamus may indeed be impacted earlier in AD [57]. Given the protracted time course of pathological changes that occur over decades in AD, these findings suggest that the hypothalamus may be implicated in AD prior to clinical diagnosis.

Based on the evidence reviewed above, the goal of the present study was to determine whether soluble amyloid pathology is present in the hypothalamus of female and male TgF344-AD rats during the earliest stages of AD development and whether it is associated with changes in peripheral energy homeostasis. If the findings indicated that hypothalamic Aβ in these rats is associated with a propensity toward weight gain and metabolic disturbances, then this might suggest that disruptions in peripheral energy homeostasis are an early symptom of AD development that may potentially contribute to the risk effects of obesity during prodromal AD.

## Materials and Methods

### Animals

A breeding colony was established at Georgia State University using hemizygous TgF344-AD sires and Fischer 344 WT dams (Charles River Laboratories, Kingston, NY). Pups were genotyped using ear tissue punch biopsies taken at weaning on postnatal (P) days 21–25 (Transnetyx, Memphis, TN). Unless otherwise stated, rats were housed in pairs or triads in ventilated polycarbonate cages in a temperature and humidity-controlled facility (25 °C, 40% humidity) on a 12-h light–dark cycle with lights on at 7 a.m. The rats were given ad libitum access to food and water. All animal procedures were approved by the Georgia State University Institutional Animal Care and Use Committee (IACUC), carried out in accordance with NIH (National Research Council) Guide for the Care and Use of Laboratory Animals, and in compliance with ARRIVE (Animal Research: Reporting of In Vivo Experiments) guidelines. The age of the TgF344-AD rats was used to determine which AD stage they were in when a measure was collected. Many were obtained in rats that were ~ 4.5 months of age, which corresponds to the preclinical stage where AD pathology and behavioral changes are present but cognitive symptoms are largely absent [25, 33, 53, 58, 59].

### Body Mass

To determine when differences in body mass emerge, male and female offspring of each genotype were weighed weekly from weaning on P21 until P70. An independent cross-sectional assessment of body mass in male and female TgF344-AD and WT rats of different age groups was also obtained from a colony at Emory University. Those procedures were approved by the Emory University IACUC.

### Energy Intake

To assess energy intake, rats were housed individually at 4–5 months of age in cages equipped with food hoppers hanging from scales (TSE LabMaster Metabolic Research Platform, TSE Systems International, Chesterfield, Missouri). This is the age when we have observed weight differences between TgF344-AD and WT in our previous research [53]. At this age, TgF344-AD rats are in the preclinical stage wherein they demonstrate brain pathology and behavioral changes, but cognition appears intact [25, 33, 53, 58, 59]. The rats were given free access to standard chow (LabDiet 5001, Richmond, IN), water, and a Nylabone for enrichment. The rats were given 3 days to acclimate to the new housing, and then, chow intake was assessed for 4 days. On the eighth day, all rats were given a palatable high calorie (kcal) HFHS diet that supplied 45% of the kcal from fat and half of the carbohydrates from sucrose (S. Table 1, Research Diets D12451, New Brunswick, NJ). Intake was assessed for 4 days yielding within-subject responses to both chow and the HFHS diet. We elected to assess intake over 4 days based on evidence showing that this duration is sufficient to assess steady-state intake. Specifically, food intake in laboratory rats is generally stable across 4 days, intake is representative by the third day, and rats adjust and stabilize their intake within 2–3 days when feeding conditions change [60, 61]. Moreover, our results show that intake is stable after 4 days in WT rats (S. Figure 2a,b). Food intake was recorded every 27 min. Each morning, the recording was stopped at 10:00 a.m. for 30 min while rats were weighed; bedding was changed as necessary, and food hoppers/water bottles were refilled. Grams of food consumed were converted to kcal consumed based on caloric content of the diet (S. Table 1). To control for the effects of differences in body mass on intake, daily energy intake was normalized to the body mass recorded at the start of that day. Energy intake was analyzed separately during the light and dark phase of the light cycle.

### Body Temperature

Unpublished body temperature data obtained from rats used in the Anderson et al. [53] study were analyzed to determine whether reductions in energy expenditure could account for the increases in body mass [53]. At 4 months of age, rats were surgically implanted with iButton temperature sensors (ThermoChron, Sidney, Australia) into the intraperitoneal cavity under deep isoflurane anesthesia (5% for initiation, 2% for maintenance, O_2_ flow rate 1.5 L/min). The rats were given carprofen (5 mg/kg SC) prior to surgery and 24 h post-surgery. The iButtons were programmed to record temperatures starting ~ 2 weeks after implantation at 30-min increments with 0.0625°C precision, encased in histology-grade paraffin wax, and sterilized with ethylene oxide gas. After 2 weeks of recovery, half of the rats in each group were randomly selected and placed on the HFHS diet. After 11 weeks of recording (7–7.5 months of age), the rats were euthanized as described in Anderson et al. [53], the iButtons were retrieved, and the temperature data were extracted [53]. Temperature readings were binned by 30 min and aligned within ± 12 min to the zeitgebers of lights-on and lights-off (e.g., the 7 a.m. timepoint represents 6:48 a.m.–7:12 a.m.). The average measurement for each timepoint was taken across 8 weeks of recording.

### Western Blot Analysis of iBAT

Western blot (Bio-Rad) was used to measure UCP1 protein expression in iBAT tissue collected from the rats in our previous study [53]. Interscapular brown adipose tissue was excised, carefully cleared of any white adipose or connective tissue, weighed, frozen in liquid nitrogen, and stored in $-$80 °C until further analyses. Western blots followed standard protocols as previously described. Protein was extracted from iBAT using radioimmunoprecipitation assay lysis buffer (RIPA) containing a protease inhibitor cocktail (Sigma-Aldrich P8340) and phosphatase inhibitors (50 mM sodium fluoride, 1 mM sodium orthovanadate, 2.5 mM sodium pyrophosphate decahydrate, and 10 mM 2,3-bisphosphoglyceric acid). Total protein concentration was determined with a detergent compatible protein assay and a microplate reader (Synergy HT, BioTek). Proteins were separated by 10% SDS-PAGE electrophoresis and transferred to a polyvinylidene difluoride membrane (Bio-Rad 10026933). Membranes were blocked in 5% nonfat dry milk for 1 h at room temperature followed by incubation in primary antibody overnight at 4°C. Membranes were then washed and incubated with secondary antibodies (Mouse-JL515035003 or Rabbit-CS7074S) at room temperature for 1 h. Protein expression was visualized using the Bio-Rad ChemiDoc Imaging System. UCP1 band density was normalized to total lane protein (trichloroethanol, Sigma-Aldrich T54801). The total amount of UCP1 was calculated as the UCP1 protein per microgram of iBAT protein multiplied by the total amount of protein present in the iBAT (see S. Table 3) [62, 63].

### EchoMRI

A separate group of rats (~ 4.5 months old) was matched for body weight within each genotype and sex and assigned to either continue standard chow (S. Table 1, LabDiet 5001, Richmond, IN) or was switched to the HFHS diet (S. Table 1, Research Diets D12451, New Brunswick, NJ) and weighed weekly. After 8 weeks on diet, body composition was assessed using an EchoMRI-700 Whole Body Analyzer (EchoMRI™, Houston, TX; Fig. 3a). Unanesthetized rats were guided into a ventilated plastic tube that was then inserted into the machine. Fat mass and lean body mass were averaged from duplicate 3-min readings. Measurements were calibrated to two known standard volumes of vegetable oil that were established through triplicate 3-min readings.

### Glucose Tolerance Test

Fasting glucose tolerance was assessed in a random subset of these rats after 9 weeks (~ 6.5 months of age) on the diet. Food was removed between 1 and 3 h after the start of the light cycle, and rats were brought into the testing room to acclimate for 8 h ± 30 min prior to the first blood glucose collection. A small puncture was made on the tip of the tail using a disposable 28-gauge lancet (CVS Thin Lancets, CVS 235157), and a drop of blood was collected to determine baseline blood glucose levels using a glucose meter (CVS Advanced Glucose Meter 968874). Then, rats were given an injection of glucose (1.7 g/kg lean mass, IP) and returned to their home cages. Lean body mass from the EchoMRI conducted 1 week earlier was used to calculate dosing. Blood samples were collected 5, 10, 30, 45, 60, 90, and 120 min following the injection by milking the tail, forcing blood to displace the scab on the tail puncture. The first drop of blood was removed with a tissue, and the subsequent drop was sampled.

### Tissue Collection

After 11 weeks on their diets (7–7.5 months of age), rats were euthanized with sodium pentobarbital (Euthasol, 150 mg/kg IP, Virbac VINV-CIII-0001). At this age, TgF344-AD rats are considered to be in the prodromal stage of AD development [37, 44, 64]. In support, we have shown previously that TgF344-AD rats fed the chow diet and WT rats fed the HFHS diet for the same 11-week period do not demonstrate memory deficits and, importantly, that TgF344-AD rats fed the HFHS diet for this 11-week period are significantly impaired. These findings suggest that the HFHS diet accelerated AD progression toward the clinical period [53]. The distance from the nose to the anus was measured to determine body length. iBAT, liver, heart, kidneys, and adrenal glands were harvested and weighed. Brains were extracted from the skull, the hypothalamus was gross-dissected, and then the brain was hemisected. The cortex was separated from the hippocampus, midbrain, thalamus, and striatum, and then, the olfactory bulb, olfactory tubercle, and a minuscule portion of frontal cortex were removed. The hypothalamus and remaining cortex were frozen on dry ice and stored at − 80 °C for further analyses.

### ELISA Analysis of Soluble Aβ

The hypothalamic samples were suspended in 20 volumes and the cortical samples in 10 volumes of cell lysis buffer (10 × complete cell lysis buffer diluted to 1 ×, Cell Signaling Technology 9803) with 1% protease and phosphatase inhibitor cocktail (HALT, Thermo Fisher 78440). The samples were then homogenized on wet ice with either 20 strokes of a mortar and pestle for hypothalamus or using a homogenizer (Pro Scientific 01–01200) for 10 s for cortex. After 30 min, the lysate was centrifuged at 16,000 × *g* for 30 min at 4 °C. The supernatant was then removed and frozen on dry ice until further analyses. Protein content was quantified by bicinchoninic acid assay (Pierce BCA; Thermo Fisher 23225), and then, Aβ species were quantified using commercial ELISA kits selective for human Aβ_42_ (Thermo Fisher KHB3441) and human Aβ_40_ (Thermo Fisher KHB3481) following the manufacturer’s instructions. The samples were quantified using a four-parameter logistical regression (myassays.com) and normalized to the total protein concentration of the sample.

### Statistics

Experimenters were blinded to the condition of the animals and samples during data collection for in vivo and post-mortem assessments. The data were tested for normality, homogeneity of variance, and covariance using R. For data that were normally distributed, values exceeding 2.5 standard deviations from the mean were identified as outliers and excluded from subsequent statistical analyses (S. Table 2). For data that were not normally distributed, values exceeding 2.5 times the interquartile range (IQR) were identified as outliers. Since litter size influences a variety of metabolic and morphometric measures [65, 66], a mixed linear model (Kenward–Roger approximation) with litter size included as a random effect was used to analyze intake with sex, diet, genotype, and time as factors (S. Table 3). When there were main effects of sex or interactions between sex and genotype, diet, or time, a subsequent mixed linear model was conducted separately in males and females with only genotype and diet (or genotype, diet, and time) as fixed effects. Effect sizes are presented as partial eta squared (${\eta}_{p}^{2}$). Pairwise *t*-tests were conducted on estimated marginal means using Bonferroni correction. Total area under the curve (tAUC) was calculated for the length of the glucose tolerance test (GTT) using the trapezoidal rule [67]. The GTT was then broken down into 30-min segments, and an AUC was taken for each segment (0–30 min, 30–60 min, 60–90 min, 90–120 min). Significant interactions between genotype and diet within each sex were analyzed with Bonferroni-corrected pairwise *t*-tests. The Aβ ELISA data were analyzed using a two-way ANOVA (sex × diet). Pearson’s correlations were computed between hypothalamic Aβ_42_ or Aβ_40_ and iBAT mass or tAUC and between iBAT UCP1 and terminal body mass. A probability of *p* < 0.05 was considered statistically significant. Averages $\pm$ standard error of the mean (SEM) are plotted for the depicted groups.

## Results

### Male and Female TgF344-AD Rats Weigh More Than WT Rats Across Several Ages During the Prodromal Period

**Fig. 1:**
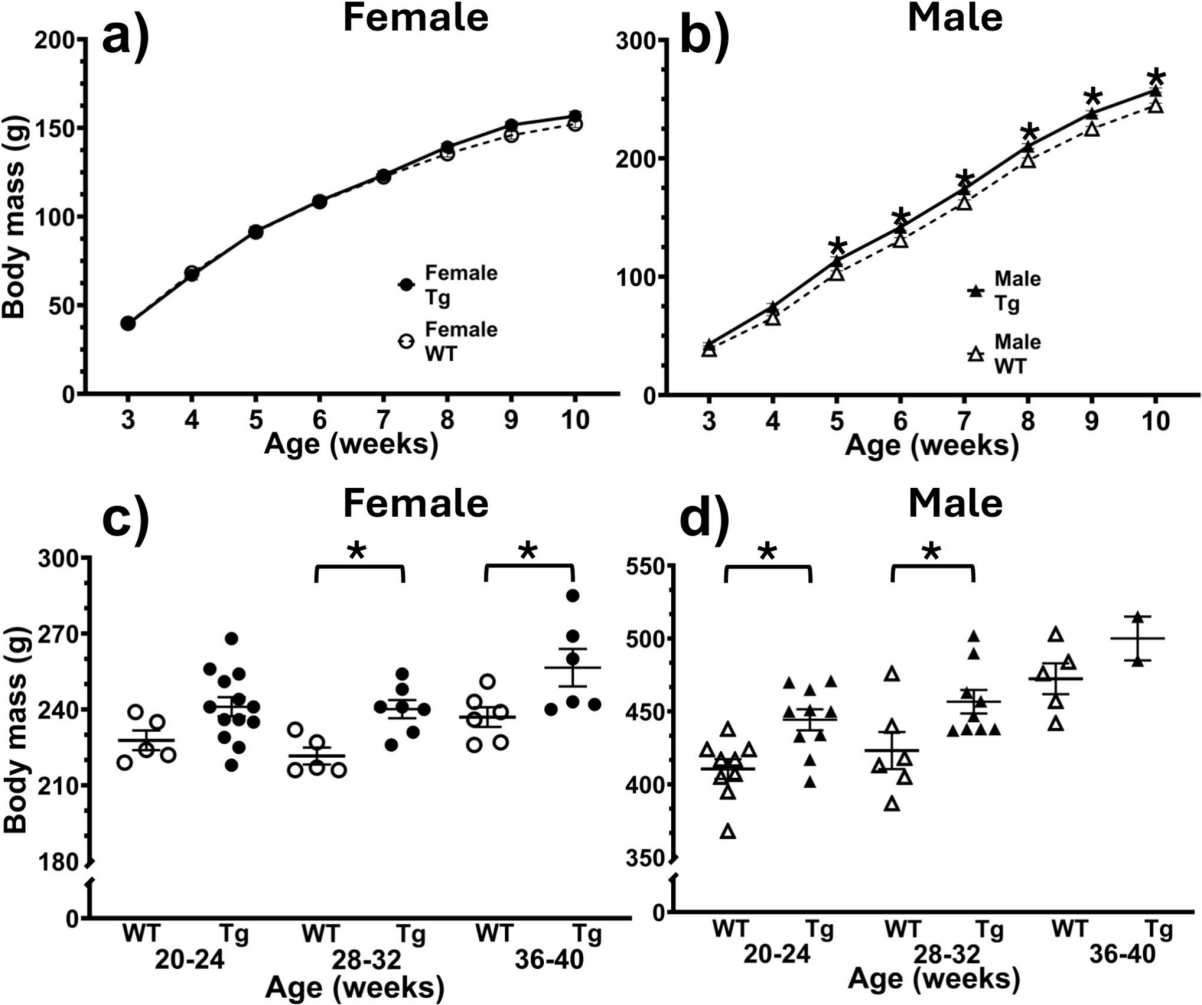
Male and female TgF344-AD rats weigh more than WT controls throughout most of the prodromal period. Male TgF344-AD rats begin to weigh more than WT controls during adolescence. **a** Female TgF344-AD rats did not display any differences during the first 10 weeks of life (*n* = 23–38/gp). **b** Male TgF344-AD rats began to weigh more than WT controls at 5 weeks of age (*n* = 23–38/gp). **c** Female TgF344-AD rats weighed significantly more than WT littermate controls at 28–32 and 36–40 weeks of age (*n* = 5–13/gp). **d** Male TgF344-AD rats weighed significantly more than WT littermate controls at 20–24 and 28–32 weeks of age (*n* = 2–12/gp). WT = wild-type, Tg = TgF344-AD, * = *p* < 0.05

Rats were weighed weekly starting after weaning (P21–P24) to identify when the weight of TgF344-AD rats begins to diverge from their WT counterparts (Fig. 1a, b). Female TgF344-AD rats did not significantly differ from WT female rats during this period and planned post hoc comparisons failed to identify any genotypic effects (Fig. 1a). For males, there was a significant interaction between genotype and age (*F*_1,567_ = 19.79, *p* = 9.68 × 10^–6^, ${\eta}_{p}^{2}$ = 0.02; Fig. 1b), such that male TgF344-AD rats had higher body mass than male WT rats (*F*_1,567_ = 56.17, *p* = 2.56 × 10^–13^, ${\eta}_{p}^{2}$ = 0.09). Planned post hoc comparisons showed that, by week 5, male TgF344-AD rats weighed significantly more than male WT rats (*t*_560.5_ = 2.845, *p* = 0.032).

A cross-sectional analysis of body mass from a different cohort of TgF344-AD rats of multiple ages showed that female and male TgF344-AD rats weighed more than their WT counterparts (female: *F*_1,36_ = 18.377, *p* = 0.000129, ${\eta}_{p}^{2}$ = 0.338, Fig. 1c; male: *F*_1,34_ = 12.44, *p* = 0.0012, ${\eta}_{p}^{2}$ = 0.268, Fig. 1d). Planned post hoc comparisons indicated that female TgF344-AD rats began to weigh significantly more than female WT rats at 28–32 weeks of age (*t* = 2.58, df = 34, *p* = 0.042); meanwhile, the male TgF344-AD rats already weighed significantly more than male WT rats by 20–24 weeks of age (*t* = 2.59, df = 36, *p* = 0.042).

### Female TgF344-AD Rats Eat More Than WT Females

**Fig. 2:**
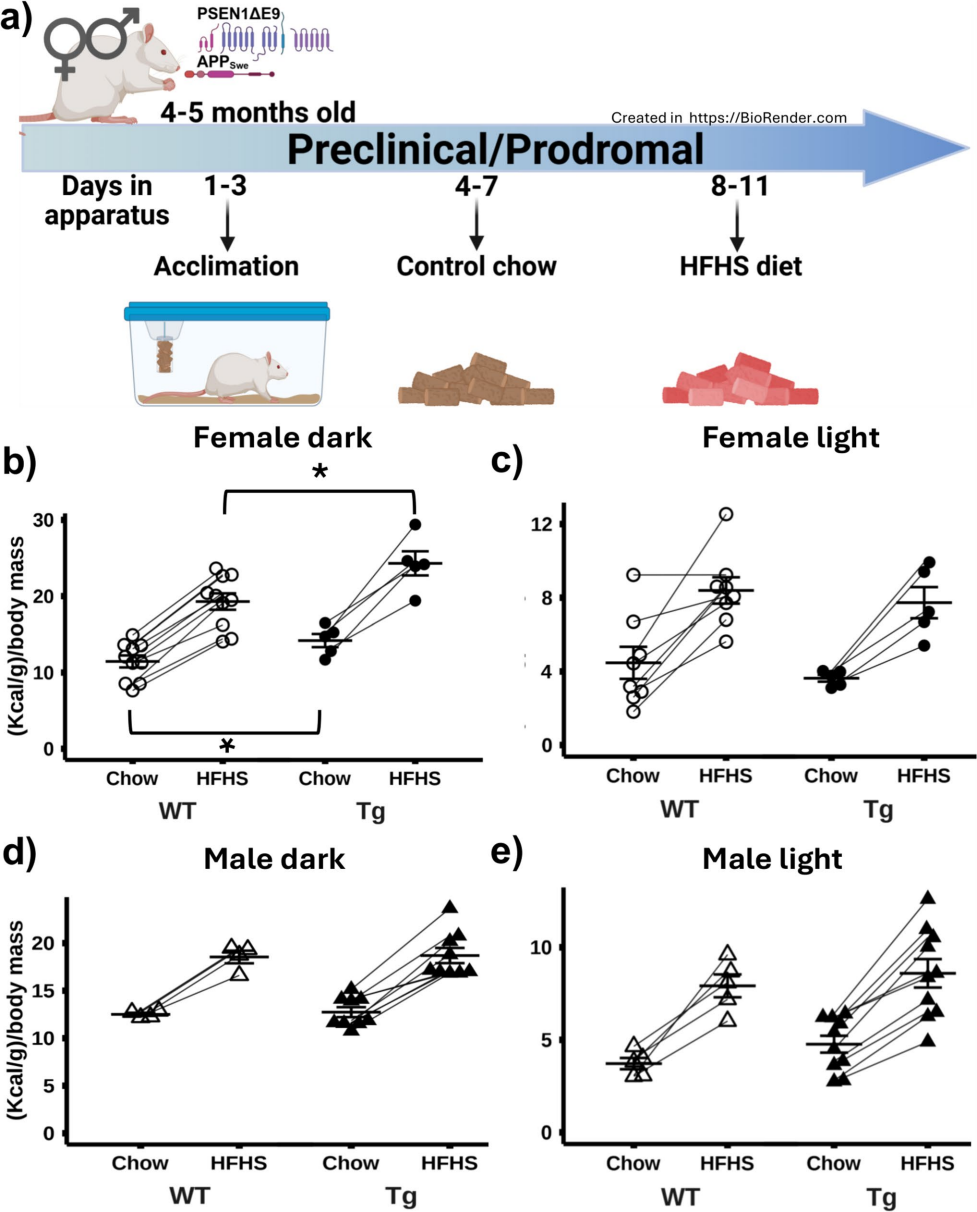
Female TgF344-AD rats consume more calories than WT female rats during the dark phase of the light cycle. **a** Experimental timeline. **b** During the dark phase, female TgF344-AD rats consumed more of the control and HFHS diet than their WT counterparts (WT: *n* = 10, Tg: *n* = 5). **c** There were no significant effects of genotype on intake during the light phase in females (WT: *n* = 8, Tg: *n* = 5). **d** There were no significant effects of genotype on intake during the dark (WT: *n* = 4, Tg: *n* = 9) or **e** light in male rats (WT: *n* = 5, Tg: *n* = 10). WT = wild-type, Tg = TgF344-AD, HFHS = high-fat high-sugar diet, * = *p* < 0.05

Energy intake was assessed in the rats from Fig. 1 when they were 4.5–5 months of age (Fig. 2a). The number of kcals consumed was divided by body mass to control for the effects of body mass on energy intake. There was a significant interaction between genotype and light cycle for energy intake (*F*_1,229.6_ = 11.09, *p* = 0.001, ${\eta}_{p}^{2}$ = 0.05) such that female TgF344-AD rats consumed more calories than female WT rats during the dark cycle (*F*_1,93.199_ = 32.854, *p* = 1.215 × 10^–7^, ${\eta}_{p}^{2}$ = 0.26; Fig. 2b; S. Figure 1a). This effect in females was accompanied by an interaction with diet (*F*_1,93.001_ = 4.754, *p* = 0.0318, ${\eta}_{p}^{2}$ = 0.05), such that there was a greater mean difference in intake between WT and TgF344-AD rats on the HFHS diet (*t*_229.91_ = 3.98, *p* = 9.24 × 10^–5^) than on the chow diet. Female TgF344-AD rats did not have elevated intake during the light phase (Fig. 2c), and intake did not differ between male WT and TgF344-AD rats during either the light or dark portion of the cycle (Fig. 2d, e; S. Figure 1b).

### Chronic Consumption of a HFHS Diet Produces Larger Increases in Body Weight in Female TgF344-AD Rats Than WT Rats

**Fig. 3:**
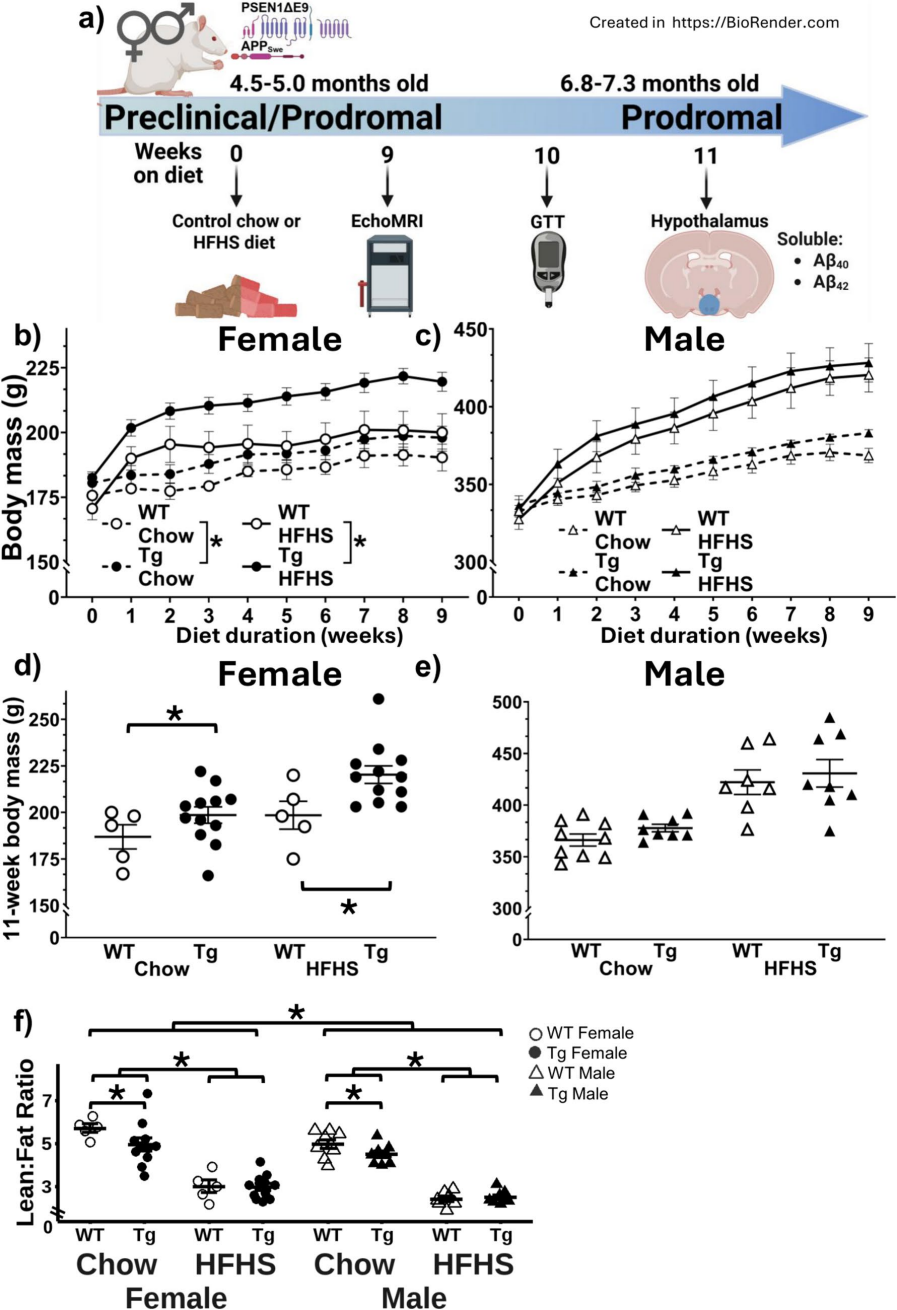
Female TgF344-AD rats accrue more body mass than WT females, especially on the HFHS diet. **a** Experimental timeline. **b** Body mass was elevated in female TgF344-AD rats fed the chow or HFHS diet (WT-Chow: *n* = 5, WT-HFHS: *n* = 5, Tg-Chow: *n* = 11, Tg-HFHS: *n* = 11). **c** This effect was not seen in male TgF344-AD rats (WT-Chow: *n* = 9, WT-HFHS: *n* = 7, Tg-Chow: *n* = 7, Tg-HFHS: *n* = 8). **d** Female TgF344-AD rats weighed more than WT rats at the end of the experiment (WT-Chow: *n* = 5, WT-HFHS: *n* = 5, Tg-Chow: *n* = 10, Tg-HFHS: *n* = 11), **e** but male TgF344-AD rats did not (WT-Chow: *n* = 9, WT-HFHS: *n* = 7, Tg-Chow: *n* = 8, Tg-HFHS: *n* = 8). **f** Chow-fed TgF344-AD rats had a lower lean-to-fat mass ratio than their WT counterparts regardless of sex (male: WT-Chow: *n* = 9, WT-HFHS: *n* = 7, Tg-Chow: *n* = 8, Tg-HFHS: *n* = 8; female: WT-Chow: *n* = 5, WT-HFHS: *n* = 5, Tg-Chow: *n* = 11, Tg-HFHS: *n* = 12). WT = wild-type, Tg = TgF344-AD, HFHS = high-fat high-sugar diet, * = *p* < 0.05

A different group of rats was placed on the HFHS diet for 11 weeks to assess the impact of chronic consumption of the diet (Fig. 3a). There was a significant main effect of genotype in female rats (*F*_1,146.5_ = 6.11, *p* = 1.46 × 10^–2^, ${\eta}_{p}^{2}$ = 0.04; Fig. 3b), with TgF344-AD rats weighing more regardless of diet. Genotype did not affect body mass in male rats during the study period (Fig. 3c). Female (*t* = 2.47, *p* = 0.015) but not male TgF344-AD rats weighed more than WT rats at the time of euthanasia (Fig. 3d, e). The higher body mass in female rats was not due to increased body size (S. Table 3).

### Chow-Fed TgF344-AD Rats Have a Reduced Lean-to-Fat Mass Ratio Regardless of Sex

Quantitative magnetic resonance results showed that diet and genotype interacted (*F*_1,56.01_ = 4.22, *p* = 0.0447, ${\eta}_{p}^{2}$ = 0.07) so that chow (*t* = 2.74, df = 53.47, *p* = 0.0333) but not HFHS diet–fed (*t* = 0.238, df = 56.95, *p* = 0.81) TgF344-AD rats had a lower lean-to-fat ratio than their WT counterparts regardless of sex. Irrespective of genotype, female rats had a higher lean-to-fat ratio than male rats (*F*_1,56.28_ = 9.75, *p* = 0.003, ${\eta}_{p}^{2}$ = 0.15; Fig. 3f) and the HFHS diet reduced the lean-to-fat ratio in both sexes (male: *F*_1,18.59_ = 2.56, *p* = 2.56 × 10^–10^, ${\eta}_{p}^{2}$ = 0.89; female: *F*_1,28.17_ = 69.03, *p* = 4.64 × 10^–9^, ${\eta}_{p}^{2}$ = 0.71).

Regardless of sex, HFHS-fed rats had increased percent body fat (*F*_1,56.79_ = 237.31, *p* = 4.84 × 10^–22^, ${\eta}_{p}^{2}$ = 0.81; S. Table 3) but there was no effect of genotype on adiposity (*F*_1,56.80_ = 0.71, *p* = 0.403, ${\eta}_{p}^{2}$ = 0.01). HFHS-fed rats also had reduced percent lean mass (*F*_1,54.26_ = 156.92, *p* = 3.70 × 10^–18^, ${\eta}_{p}^{2}$ = 0.74; S. Table 3). The impact of diet on percent lean mass depended on sex (*F*_1,56_ = 6.42, *p* = 0.014, ${\eta}_{p}^{2}$ = 0.10). Specifically, post hoc analyses showed that diet and genotype interacted in males (*F*_1,23.16_ = 7.41, *p* = 0.012, ${\eta}_{p}^{2}$ = 0.31) but not females (*F*_1,28.46_ = 0.84, *p* = 0.368, ${\eta}_{p}^{2}$ = 0.03) such that chow-fed TgF344-AD males had less percent lean mass than chow-fed WT males (*t* = 3.03, df = 22.15, *p* = 0.017).

### Male TgF344-AD Rats Fed the HFHS Diet Have Impaired Glucose Regulation

**Fig. 4:**
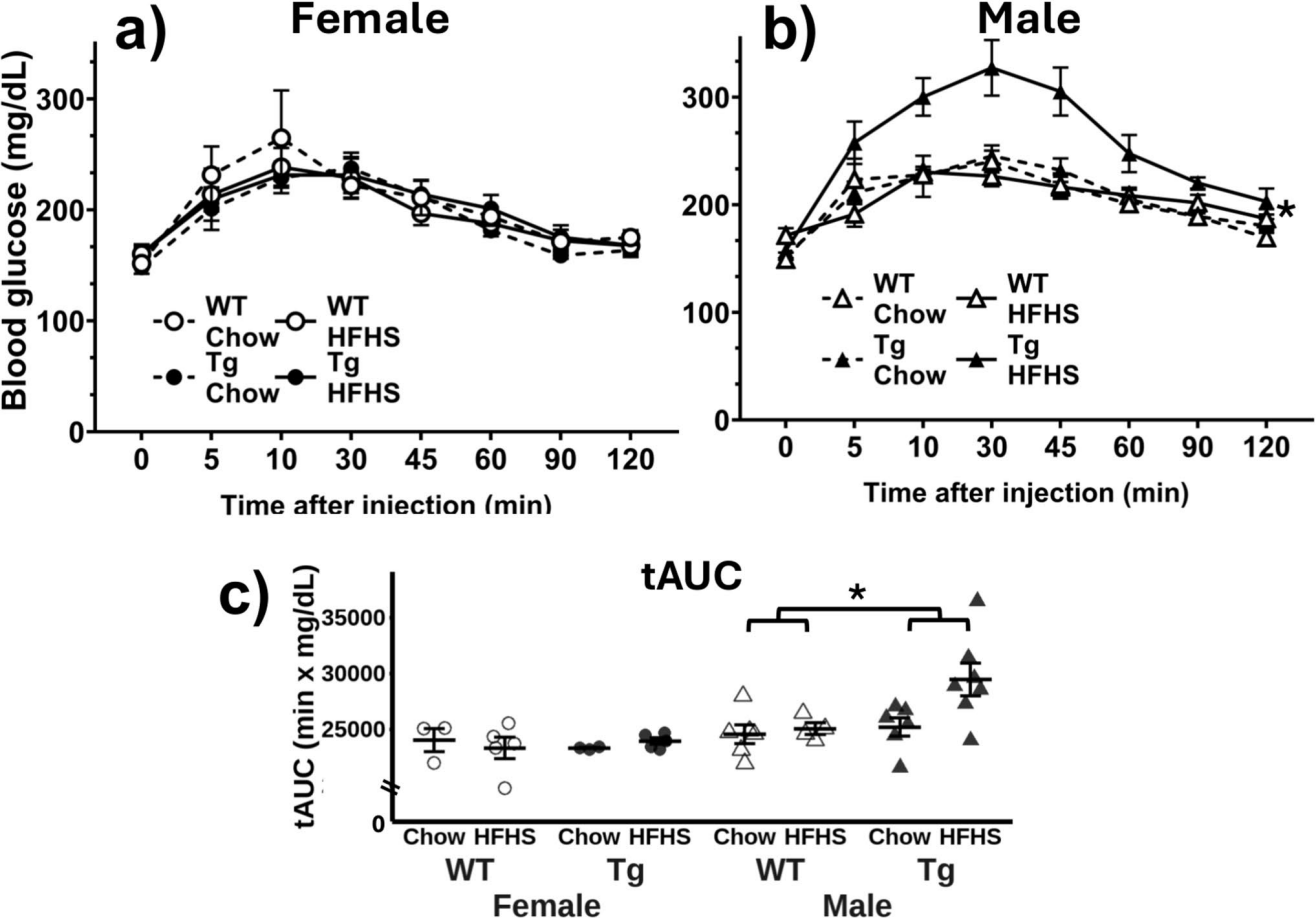
Male but not female TgF344-AD rats have disrupted glucose homeostasis. **a** Female rats (6.3–7 months) did not exhibit diet- or genotype-induced disruptions in glucose regulation (WT-Chow: *n* = 3, WT-HFHS: *n* = 5, Tg-Chow: *n* = 4, Tg-HFHS: *n* = 5). **b** Glucose administration increased blood glucose levels more in male TgF344-AD rats fed the HFHS diet than any other group of male rats (WT-Chow: *n* = 6, WT-HFHS: *n* = 4, Tg-Chow: *n* = 6, Tg-HFHS: *n* = 7). c Male TgF344-AD rats had elevated tAUC (male: WT-Chow: *n* = 6, WT-HFHS: *n* = 4, Tg-Chow: *n* = 6, Tg-HFHS: *n* = 7; female: WT-Chow: *n* = 3, WT-HFHS: *n* = 5, Tg-Chow: *n* = 3, Tg-HFHS: *n* = 5). WT = wild-type, Tg = TgF344-AD, HFHS = high-fat high-sugar diet, * = *p* < 0.05

Glucose administration significantly increased blood glucose concentrations in all groups (*F*_1,486.41_ = 17.46, *p* = 1.12 × 10^–20^, ${\eta}_{p}^{2}$ = 0.20; Fig. 4a, b). There was a significant interaction between genotype and diet (*F*_1,439.78_ = 5.78, *p* = 0.017, ${\eta}_{p}^{2}$ = 0.01) that was specific to males (*F*_1,221.85_ = 5.47, *p* = 0.02, ${\eta}_{p}^{2}$ = 0.17; females *F*_1,193.05_ = 1.14, *p* = 0.287, ${\eta}_{p}^{2}$ = 0.01). Specifically, glucose administration produced larger increases in blood glucose concentrations in HFHS-fed male TgF344-AD than WT males fed the same diet (*t* = 3.57, df = 230.53, *p* = 0.002; Fig. 4b). When the AUC was broken down into 30-min segments, there was an interaction between sex and genotype from 0 to 30 min (*F*_1,31.8_ = 4.61, *p* = 0.039, ${\eta}_{p}^{2}$ = 0.13; Table 1) and main effects of sex from 30 to 60 min (*F*_1,28.85_ = 5.47, *p* = 0.026, ${\eta}_{p}^{2}$ = 0.15; Table 1), 60–90 min (*F*_1,20_ = 17.89, *p* = $4.11 \times {10}^{-4}$, ${\eta}_{p}^{2}$ = 0.47; Table 1), and 90–120 min (*F*_1,20_ = 14.73, *p* = 0.001, ${\eta}_{p}^{2}$ = 0.42; Table 1). During the post-injection incline from 0 to 30 min (Table 1), diet and genotype interacted in male rats (*F*_1,16.31_ = 5.32, *p* = 0.035, ${\eta}_{p}^{2}$ = 0.25). Specifically, HFHS-fed TgF344-AD rats had a greater AUC than their HFHS-fed WT counterparts (*t* = 3.51, df = 16.23, *p* = 0.011) and chow-fed TgF344-AD rats (*t* = 2.92, df = 16.52, *p* = 0.039). As blood sugar levels declined from 30 to 60 min (Table 1), diet and genotype interacted in male rats (*F*_1,15.58_ = 4.69, *p* = 0.046, ${\eta}_{p}^{2}$ = 0.23), such that HFHS-fed male TgF344-AD rats had a greater AUC than male WT rats fed the same diet (*t* = 3.44, df = 15.58, *p* = 0.014). HFHS-fed male rats had a greater AUC than their male chow-fed counterparts as blood glucose levels continued to decline between 60 and 90 min (*F*_1,9.91_ = 7.16, *p* = 0.023, ${\eta}_{p}^{2}$ = 0.42; Table 1) and 90–120 min (*F*_1,10.16_ = 7.68, *p* = 0.019, ${\eta}_{p}^{2}$ = 0.43; Table 1). TgF344-AD males had a greater tAUC than WT males (*F*_1,15.68_ = 4.67, *p* = 0.047, ${\eta}_{p}^{2}$ = 0.23; Fig. 4c), but genotype did not interact with HFHS diet to further increase AUC (*F*_1,15.68_ = 2.72, *p* = 0.119, ${\eta}_{p}^{2}$ = 0.15).

**Table 1:** Fasting glucose and glucose tolerance test

	Female				
	Chow		HFHS		Significant effects
	WT	Tg	WT	Tg	
Fasting blood glucose (mg/dL)	151.25 ± 0.95	153.33 ± 5	160.2 ± 6.44	160.43 ± 8.83	a
0–30min AUC (min × mg/dL)	7064.17 ± 688.44	6848.33 ± 351.11	6734.5 ± 482.69	6719.5 ± 65.72	b
30–60 min	6825 ± 356.34	6577.5 ± 256.14	6073.5 ± 306.31	6432 ± 115.16	b
60–90 min	5480 ± 135.37	5095 ± 114.35	5397 ± 216.15	5616 ± 130.65	
90–120 min	5195 ± 108.28	4780 ± 21.79	5097 ± 279.31	5210 ± 5	
	Male				
Fasting blood glucose (mg/dL)	149.71 ± 0.68	149.57 ± 3.68	170.2 ± 5.44	156.29 ± 4.96	a
0–30min AUC (min × mg/dL)	6738.08 ± 258.82	6927.5 ± 320.79	6531.25 ± 67.15	7976.25 ± 192.39	b, c
30–60 min	6580 ± 271.6	6893.75 ± 316.27	6510 ± 225.94	8053.75 ± 232.75	b, c
60–90 min	5847.5 ± 174.65	6907.5 ± 234.7	6153.75 ± 191.15	6887.14 ± 210.77	d
90–120 min	5375 ± 174.01	5462.5 ± 201.52	5831.25 ± 191.64	6261.43 ± 165.74	d

Effects: a: Significant main effect of Diet regardless of sex (*p* < .05); b: Significant interaction between sex and genotype (*p* < .05); c: Significant interaction between Diet and Genotype in males (*p* < .05); d: Significant effect of HFHS diet in males (*p* < .05).

Male WT-Chow: *n* = 6–7, Tg-Chow: *n* = 6–7, WT-HFHS: *n* =4–6, Tg-HFHS: *n* = 7–8; Female: WT-Chow: *n* = 3–4, Tg-Chow: *n* =3–9, WT-HFHS: *n* = 5, Tg-HFHS: *n* = 5–10

Female TgF344-AD rats had comparable AUC to their WT counterparts from 0 to 30 min (*F*_1,10.21_ = 1.75, *p* = 0.215, ${\eta}_{p}^{2}$ = 0.15; Table 1), 30–60 min (*F*_1,12.06_ = 2.48, *p* = 0.141, ${\eta}_{p}^{2}$ = 0.17; Table 1), 60–90 min (*F*_1,12.82_ = 0.001, *p* = 0.973, ${\eta}_{p}^{2}$ = $9.25 \times {10}^{-5}$; Table 1), and 90–120 min (*F*_1,12.82_ = 0.18, *p* = 0.675, ${\eta}_{p}^{2}$ = 0.01; Table 1). There was no effect of genotype (*F*_1,146.02_ = 0.417, *p* = 0.519, ${\eta}_{p}^{2}$ = 0.00285) on blood glucose concentrations or on tAUC in female rats (Fig. 4c). There was no significant effect of genotype on the baseline blood glucose concentrations after the 8-h fast (*F*_1,31.5_ = 0.1, *p* = 0.754, ${\eta}_{p}^{2}$ = 0.02; Table 1).

### iBAT and Adrenal Gland Mass Are Reduced in Female TgF344-AD Rats

**Fig. 5:**
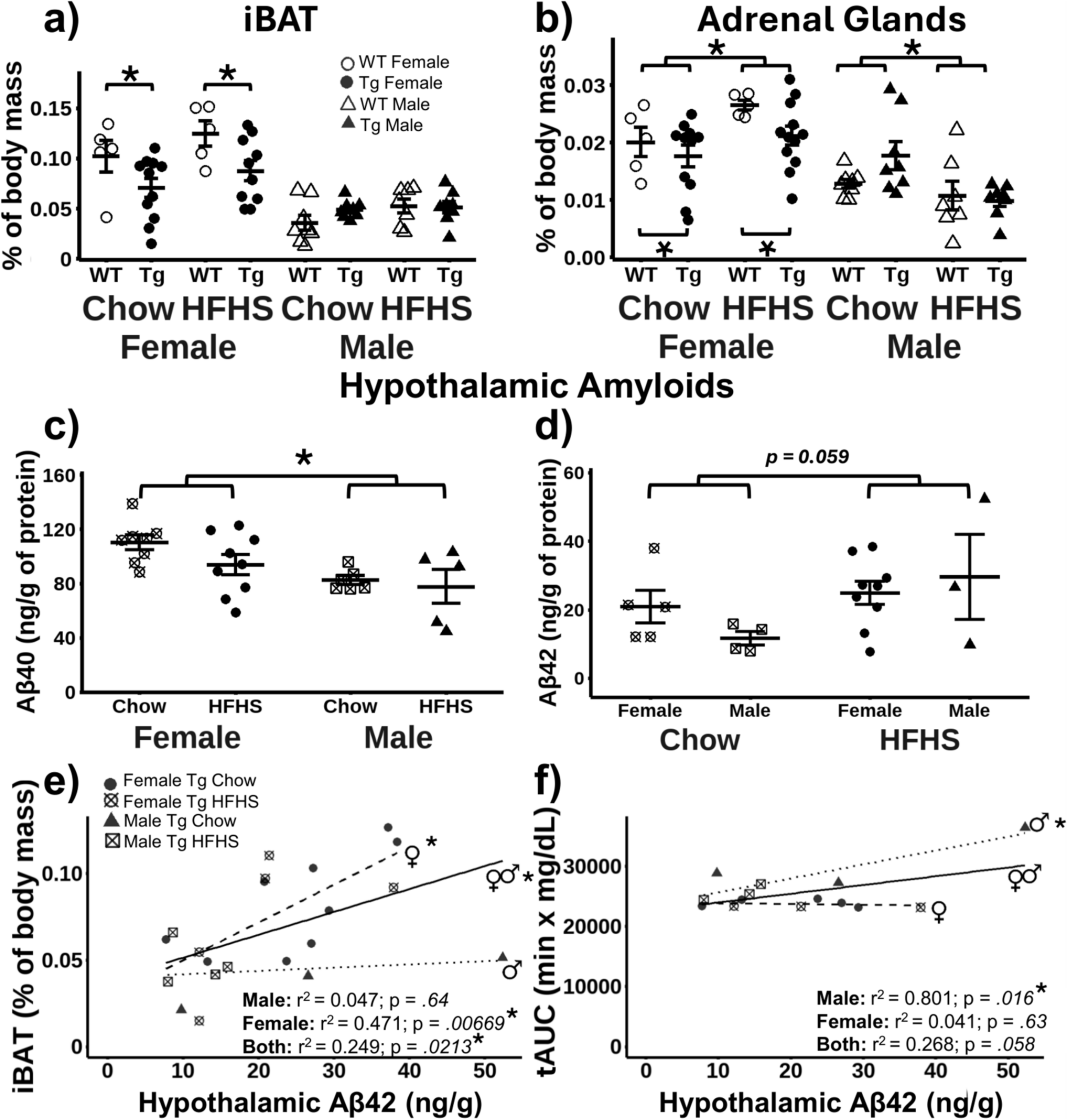
iBAT and adrenal gland mass are reduced in TgF344-AD females regardless of diet; soluble human Aβ_40_ and Aβ_42_ are expressed in the hypothalamus of prodromal TgF344-AD rats. **a** Female TgF344-AD rats had less iBAT mass than female WT rats regardless of diet (male: WT-Chow: *n* = 8, WT-HFHS: *n* = 7, Tg-Chow: *n* = 8, Tg-HFHS: *n* = 8; female: WT-Chow: *n* = 5, WT-HFHS: *n* = 5, Tg-Chow: *n* = 11, Tg-HFHS: *n* = 12). **b** Female TgF344-AD rats had reduced adrenal gland mass. HFHS diet increased adrenal mass in females and reduced it in males (male: WT-Chow: *n* = 8, WT-HFHS: *n* = 7, Tg-Chow: *n* = 8, Tg-HFHS: *n* = 8; female: WT-Chow: *n* = 5, WT-HFHS: *n* = 5, Tg-Chow: *n* = 11, Tg-HFHS: *n* = 12)*.* **c** Female TgF344-AD rats (6.8–7.3 months) had more Aβ_40_ in the hypothalamus than male rats (male: Tg-Chow: *n* = 6, Tg-HFHS: *n* = 5; female Tg-Chow: *n* = 8, Tg-HFHS: *n* = 9). **d** There was a strong trend for HFHS diet–induced increases in hypothalamic Aβ_42_ in male and female Tg rats (male: Tg-Chow: *n* = 4, Tg-HFHS: *n* = 3; female Tg-Chow: *n* = 5, Tg-HFHS: *n* = 9). **e i**BAT mass and hypothalamic Aβ_42_ were positively correlated in female TgF344-AD rats (male: *n* = 7; female: *n* = 14). **f** There was a positive association between tAUC and hypothalamic Aβ_42_ in male TgF344-AD rats (male: *n* = 6; female: *n* = 8). WT = wild-type, Tg = TgF344-AD, HFHS = high-fat high-sugar diet, * = *p* < 0.05

iBAT mass was reduced in 7-month-old female TgF344-AD rats. Sex and genotype significantly interacted to impact iBAT mass (*F*_1,51.74_ = 12.35, *p* = 9.25 × 10^–4^, ${\eta}_{p}^{2}$ = 0.19; Fig. 5a) such that female TgF344-AD rats had reduced iBAT mass compared to WT females (*F*_1,26.87_ = 13.59, *p* = 0.001, ${\eta}_{p}^{2}$ = 0.34). The decline was specific to females as iBAT depots in male WT and TgF344-AD rats were comparable (*F*_1,23.14_ = 0.88, *p* = 0.358, ${\eta}_{p}^{2}$ = 0.04).

There was a significant interaction between sex and diet (*F*_1,52.25_ = 12.45, *p* = 8.75 × 10^–4^, ${\eta}_{p}^{2}$ = 0.19) and between sex and genotype (*F*_1,54.08_ = 5.26, *p* = 0.026, ${\eta}_{p}^{2}$ = 0.09) on adrenal gland mass (Fig. 5b). Specifically, female TgF344-AD rats had lighter adrenal glands than their WT counterparts (*F*_1,27.94_ = 6.73, *p* = 0.015, ${\eta}_{p}^{2}$ = 0.19), an effect that was not observed in males (*F*_1,21.79_ = 1.58, *p* = 0.223, ${\eta}_{p}^{2}$ = 0.07). There was no impact of genotype on the mass of the heart (*F*_1,51.99_ = 1.02, *p* = 0.316, ${\eta}_{p}^{2}$ = 0.02; S. Table 3), liver (*F*_1,56.94_ = 2.08, *p* = 0.155, ${\eta}_{p}^{2}$ = 0.04; S. Table 3), or kidneys (*F*_1,50.85_ = 1.15, *p* = 0.289, ${\eta}_{p}^{2}$ = 0.02; S. Table 3).

### Soluble Aβ Is Detectable in the Hypothalamus of TgF344-AD Rats During the Prodromal Phase; Hypothalamic, but Not Cortical, Aβ_42_ Correlates with iBAT Mass in Females and Glucose Dysregulation in Males

Soluble human Aβ_40_ and Aβ_42_ were measured in the hypothalamus of 7-month-old TgF344-AD rats after 11 weeks on the diet (Fig. 3a). In a pilot experiment, we could not detect human Aβ_40_ and Aβ_42_ in WT rats, confirming that the human Aβ assays did not cross-react with endogenous amyloid species (*n* = 4, data not shown). Female TgF344-AD rats had more soluble Aβ_40_ (*F*_1,25_ = 8.423, *p* = 0.008, ${\eta}_{p}^{2}$ = 0.26; Fig. 5c) than males but they did not differ in Aβ_42_ levels (*F*_1,25_ = 0.181, *p* = 0.676, ${\eta}_{p}^{2}$ = 0.01; Fig. 5d). The HFHS diet did not affect Aβ_40_ levels in male or female TgF344-AD rats (male: *F*_1,9_ = 0.167, *p* = 0.692, ${\eta}_{p}^{2}$ = 0.018; female: *F*_1,16_ = 1.869, *p* = 0.19, ${\eta}_{p}^{2}$ = 0.105). Hypothalamic soluble Aβ_42_ was undetectable in 50% of male TgF344-AD rats (4 chow, 3 HFHS) and 25% of female TgF344-AD rats (2 chow, 2 HFHS; no significant differences between sexes *χ*^2^ = 1.0772, df = 1, *p* = 0.2993, *V* = 0.189). However, there was a strong trend for the HFHS diet to increase hypothalamic Aβ_42_ in TgF344-AD rats (*F*_1,17_ = 4.106, *p* = 0.059, ${\eta}_{p}^{2}$ = 0.195; Fig. 5d).

As we found evidence of iBAT dysregulation in female TgF344-AD rats and disturbed glucose regulation in male TgF344-AD rats, we sought to determine whether hypothalamic Aβ_42_ and/or Aβ_40_ correlated with iBAT mass and the AUCs during the GTT. When collapsed by sex, iBAT mass correlated positively with hypothalamic Aβ_42_ (*R*^2^ = 0.483, *p* = 0.0213, *α*_adj_ = 0.0167; Fig. 5e). When separated by sex, there was a positive correlation between iBAT mass and hypothalamic Aβ_42_ in female TgF344-AD rats (*R*^2^ = 0.441, *p* = 6.69 × 10^–4^, *α*_adj_ = 0.0167) but not in male TgF344-AD rats (*R*^2^ = 0.002, *p* = 0.939, *α*_adj_ = 0.0167). By contrast, hypothalamic Aβ_42_ correlated with AUC during the glucose tolerance test in male TgF344-AD rats (*R*^2^ = 0.801, *p* = 0.016, *α*_adj_ = 0.0167; Fig. 5f) but not in female TgF344-AD rats (*R*^2^ = 0.041, *p* = 0.63, *α*_adj_ = 0.0167) or when collapsed by sex (*R*^2^ = 0.268, *p* = 0.058, *α*_adj_ = 0.0167). Hypothalamic Aβ_40_ did not correlate with iBAT, tAUC, or any of the AUC segments (data not shown).

To determine whether the positive correlation between hypothalamic soluble Aβ_42_ and peripheral metabolic disturbances extends to other brain regions, we measured soluble Aβ_42_ in the cortex from the same rats. The results indicated that cortical soluble Aβ_42_ did not correlate with iBAT mass (male: *r*^2^ = 0.005, *p* = 0.816, *α*_adj_ = 0.0167; female: *r*^2^ = 0.054, *p* = 0.341, *α*_adj_ = 0.0167; S. Figure 2a) nor tAUC (male: *r*^2^ = 0.076, *p* = 0.413, *α*_adj_ = 0.0167; female: *r*^2^ = 0.016, *p* = 0.765, *α*_adj_ = 0.0167; S. Figure 2b) in TgF344-AD rats of either sex. Neither the HFHS diet (*F*_1,25_ = 0.275, *p* = 0.604, ${\eta}_{p}^{2}$ = 0.009) nor biological sex (*F*_1,25_ = 1.846, *p* = 0.184, ${\eta}_{p}^{2}$ = 0.058) affected cortical soluble Aβ_42_ levels (S. Figure 2c).

### TgF344-AD Rats Have Elevated Body Temperatures

**Fig. 6:**
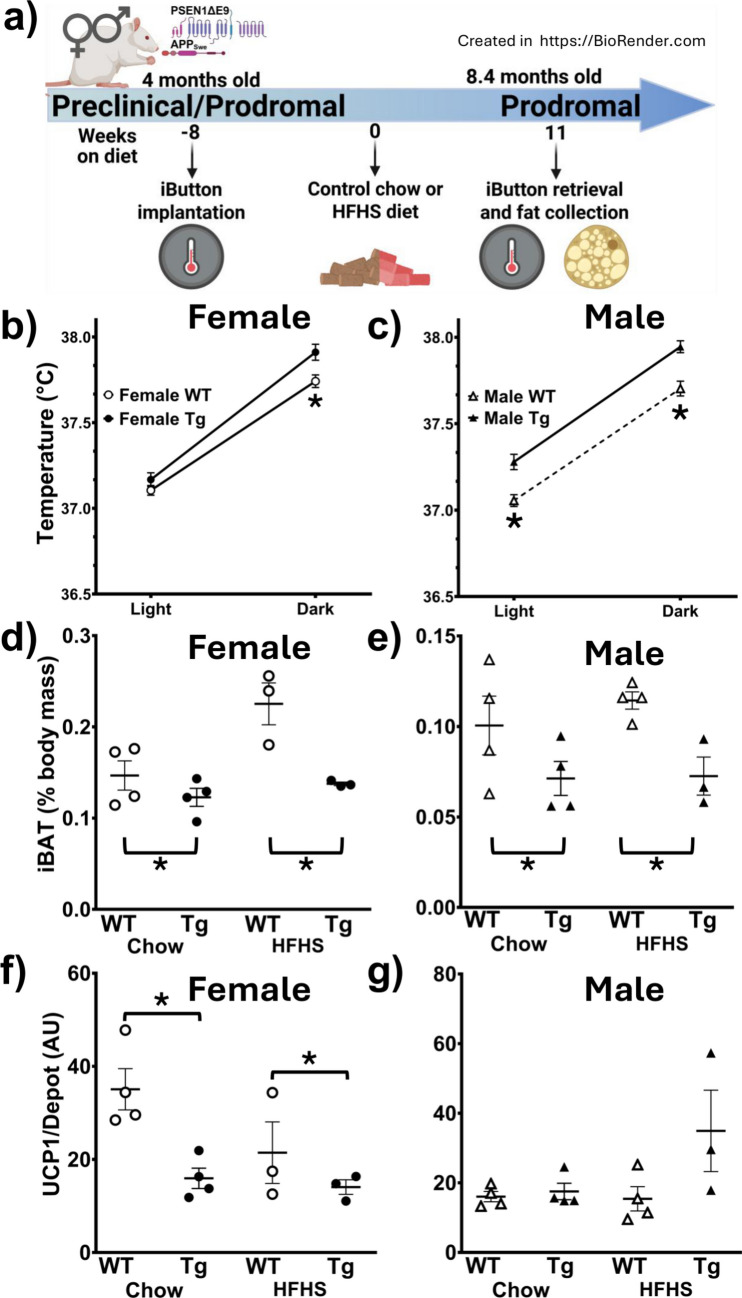
Body temperature is increased, but iBAT mass and UCP1 levels are reduced in female TgF344-AD rats. **a** Experimental timeline. **b** Female TgF344-AD rats (5–7 months of age) had higher body temperatures than WT females during the dark phase of the light cycle (WT: *n* = 13, Tg: *n* = 13). **c** Male TgF344-AD rats had higher body temperatures than WT males during both the light and dark phase (WT: *n* = 15, Tg: *n* = 14). **d** iBAT mass was reduced in female TgF344-AD rats (WT-Chow: *n* = 4, WT-HFHS: *n* = 4, Tg-Chow: *n* = 3, Tg HFHS: *n* = 3) and **e** male TgF344-AD rats (WT-Chow: *n* = 4, WT-HFHS: *n* = 4, Tg-Chow: *n* = 4, Tg HFHS: *n* = 3). **f** Female TgF344-AD rats expressed less UCP1 in iBAT tissue than WT females (WT-Chow: *n* = 4, WT-HFHS: *n* = 4, Tg-Chow: *n* = 3, Tg HFHS: *n* = 3) while **G** male TgF344-AD rats expressed UCP1 levels comparable to WT rats (WT-Chow: *n* = 4, WT-HFHS: *n* = 4, Tg-Chow: *n* = 4, Tg HFHS: *n* = 3). WT = wild-type, Tg = TgF344-AD, HFHS = high-fat high-sugar diet, * = *p* < 0.05

Body temperature was assessed between 6 and 8 months of age [53]. There was a significant interaction between genotype and light cycle for females (*F*_1,479_ = 8.4789, *p* = 0.00376, ${\eta}_{p}^{2}$ = 0.02), with female TgF344-AD rats exhibiting higher temperatures than female WT rats only during the dark phases (*t*_21.99_ = 2.49, *p* = 0.042; Fig. 6b; S. Figure 1c). For males, a main effect of genotype was observed (*F*_1,25_ = 24.31, *p* = 4.44 × 10^–5^, ${\eta}_{p}^{2}$ = 0.53), such that male TgF344-AD rats had higher body temperatures than male WT rats in both the light and dark (*t*_20.33_ = 3.46, *p* = 0.0049; Fig. 3c; S. Figure 1d). A significant interaction between genotype and light cycle (*F*_1,479_ = 5.112, *p* = 0.024, ${\eta}_{p}^{2}$ = 0.01) indicated that the temperature difference in the dark was greater than in the light in male TgF344-AD rats (S. Figure 1d).

### TgF344-AD Rats Have Reduced iBAT Mass; Female TgF344-AD Rats Also Have Reduced UCP1

At 8.4 months of age, female and male TgF344-AD rats had reduced iBAT mass (*F*_1,21_ = 24.72, *p* = 0.000064, ${\eta}_{p}^{2}$ = 0.541; Fig. 6d, e) compared to their WT counterparts. There was a significant interaction between genotype and sex for UCP1 expression (*F*_1,20_ = 11.18, *p* = 0.0032, ${\eta}_{p}^{2}$ = 0.359; Fig. 6f, g). In females, a main effect of genotype was observed (*F*_1,10_ = 10.57, *p* = 0.0087, ${\eta}_{p}^{2}$ = 0.514), such that TgF344-AD females on both diets exhibited reduced UCP1 expression (*t* = 3.252, *p* = 0.0087), whereas in males, there was a trend toward increased UCP1 in TgF344-AD rats (*F*_1,11_ = 4.227, *p* = 0.064, ${\eta}_{p}^{2}$ = 0.278). A negative association between terminal body mass and UCP1 expression was found in female rats (*r*^2^ = 0.336, *p* = 0.0299, *α*_adj_ = 0.025; S. Figure 1e), and a positive association between terminal body mass and UCP1 expression was found in male rats (*r*^2^ = 0.287, *p* = 0.0486, *α*_adj_ = 0.025; S. Figure 1e).

## Discussion

Collectively, the results of the present study show that peripheral energy homeostasis is disrupted in TgF344-AD rats and that the timing and profile of these changes depend on sex. Female but not male TgF344-AD rats display decreases in iBAT UCP1 levels, increased energy intake and body temperature during the dark phase, and HFHS diet–induced increases in body mass. Female TgF344-AD rats also exhibit decreases in iBAT mass at an earlier age than male TgF344-AD rats. Male TgF344-AD rats have increased body temperature during both the light and dark phase and only male TgF344-AD rats had HFHS diet–induced impairment of glucose clearance. Further, male TgF344-AD rats begin to outweigh their WT littermates during adolescence, at a younger age than female TgF344-AD rats. These increases in body mass persist for most of the prodromal period, particularly in female rats.

The present findings appear to be the first to show that soluble Aβ is present in the hypothalamus during the prodromal period in TgF344-AD rats. This finding is consistent with observations in AD patients showing that the hypothalamus displays amyloid plaques and undergoes neurodegeneration and atrophy during early AD [14, 57, 68] and with the finding that glucose metabolism is reduced in the hypothalamus of presymptomatic Tg2576 mice that also overexpress APP [69]. Our data also show that there is more soluble Aβ_40_ in the hypothalamus of female than male rats, although the consequences of these elevations are unclear. Interestingly, there was a trend suggesting that the HFHS diet increases hypothalamic soluble Aβ_42_ levels in TgF344-AD rats. There were also significant sex-specific positive correlations between hypothalamic Aβ_42_ and glucose dysregulation in TgF344-AD male rats and with increased iBAT mass in TgF344-AD females. By contrast, cortical soluble Aβ_42_ did not correlate with iBAT mass nor glucose AUC. Also, there was not a trend for the HFHS diet to increase cortical soluble Aβ_42_ as was observed in the hypothalamus.

Taken together, these findings show that the relationship between soluble Aβ_42_ and peripheral metabolic dysfunction is not global, raise the possibility that the association may be specific to the hypothalamus, and are consistent with the hypothesis that hypothalamic Aβ_42_ mediates the sex-dependent peripheral changes. The paraventricular nucleus of the hypothalamus (PVN) controls sympathetic outflow to BAT and projects to the lateral hypothalamus and the arcuate nucleus, which are critical for the regulation of feeding behavior, BAT thermogenesis, and blood glucose regulation [70, 71]. Separate populations of neurons in the PVN mediate BAT thermogenesis vs. peripheral glucose homeostasis [72, 73]. Since the measures of soluble Aβ in the present study were obtained from the entire hypothalamus, additional research is needed to determine whether these sex differences in metabolic disturbances are associated with differences in the hypothalamic nuclei and specific neuronal subpopulations in female vs. male TgF344-AD rats.

Our novel findings that iBAT correlates positively with hypothalamic Aβ_42_ in female rats suggest that hypothalamic dysfunction may mediate the iBAT disturbances. As female TgF344-AD rats also exhibited reduced iBAT UCP1 expression, this association may be evidence of the whitening of the iBAT and its accumulation of excess lipids [74, 75]. Indeed, decreased UCP1 significantly contributes to whitening-associated morphological changes [76] and is considered the mechanism mediating whitening [77, 78]. Relatedly, intracerebroventricular infusions of Aβ oligomers induce hypothalamic inflammation that precedes glucose intolerance [79]. Collectively, these lines of evidence suggest that soluble Aβ oligomers may induce hypothalamic pathology to induce peripheral metabolic dysfunction. The coincidence of peripheral metabolic dysfunction with AD is well established, but the presentation of specific metabolic dysfunctions is heterogeneous in the patient population [80, 81]. Our findings suggest that elevated soluble Aβ may drive some of this heterogeneity by interacting with biological sex.

Our findings mechanistically add to the growing evidence indicating that energy homeostasis is disrupted in TgF344-AD rats in a sex-dependent manner [41, 82]. Our findings also raise the possibility that increased energy intake and decreased UCP1-mediated thermogenesis contribute to these increases in weight gain in female TgF344-AD rats. This likelihood is supported by the negative correlation between iBAT UCP1 levels and body weight in female rats that we observed. Our findings show further that glucose regulation was only impaired in male TgF344-AD rats fed the HFHS diet. Even though WT rats fed the HFHS diet had comparable body mass, adiposity, and lean-to-fat ratios, they did not have disturbed glucose tolerance. This finding is consistent with observations in humans showing that many obese individuals are metabolically healthy [83]. Given that the disturbed glucose regulation was seen in TgF344-AD but not WT rats and that the disturbed glucose tolerance correlated with hypothalamic Aβ_42_, these findings suggest hypothalamic Aβ_42_ increases susceptibility to HFHS diet–induced type II diabetes risk. Compared to metabolically healthy obese individuals, obese individuals with impaired glucose regulation have more visceral fat, dysfunctional expansion of adipocytes leading to lipid spillover into the pancreas, increased proinflammatory macrophages, decreased muscle insulin sensitivity, lower adiponectin levels [84, 85], and impaired mitochondrial function [86]. Additional research is needed to determine whether any of these factors contributed to the HFHS diet–induced glucose dysregulation in male TgF344-AD rats. The finding that glucose regulation was impaired in male but not female TgF344-AD rats fed the HFHS diet is consistent with research showing that females are resilient to the metabolic impact of obesity on glucose regulation [87–90] and that type II diabetes is more prevalent in human males than females [91, 92]. The relationship between sex and glucose regulation appears to be moderated, in part, by age. Although young women are resilient [93–96], older women appear to lose this resiliency, likely due to the loss of the protective effects of ovarian hormones [97–99]. These findings lead to the prediction that female TgF344-AD rats will develop glucose intolerance as they age. In support, others have shown that older (9 and 12 months old) female TgF344-AD rats fed chow have impaired glucose regulation [41].

The present finding that adrenal gland mass is decreased in female TgF344-AD rats is inconsistent with evidence indicating that the hypothalamic–pituitary–adrenal (HPA) axis is overactivated in AD [100], given that HPA overactivation induces adrenal hyperplasia [101]. However, adrenal function has not been well studied during the transition from MCI to AD. It is therefore possible that preclinical or prodromal AD patients might display secondary or tertiary adrenal insufficiency before going on to develop HPA overactivation in later stages. Secondary and tertiary adrenal insufficiency is due to pituitary or hypothalamic dysfunction, respectively, which could reduce adrenal gland size [102].

The finding that TgF344-AD rats have higher body temperatures than WT rats is consistent with evidence showing that AD patients have increased core body temperature [103]. The increases we observed are not likely due to elevated activity levels, because home cage activity and locomotor movement and speed are not increased in male or female TgF344-AD rats across a wide range of ages and measures [25, 26, 59, 104]. The elevations in body temperature during the dark phase observed in female TgF344-AD rats are surprising because UCP1 levels were reduced in these rats. We speculate that this increase that is restricted to the dark phase is due to their elevated energy intake that also occurred only during the dark and the associated increase in activity and digestion-induced thermogenesis. Given that male TgF344-AD rats did not consume more food, did not have significant reductions in UCP1, and that UCP1 was positively associated with terminal body mass, the processes that contributed to their elevated body temperatures remain unclear. One possibility is that UCP1-independent mechanisms of thermogenesis mediate these increases in male TgF344-AD rats, such as UCP3-related nonshivering thermogenesis in skeletal muscle [53, 105].

Our findings raise the intriguing possibility that metabolic disturbances are an early symptom of AD. In humans, AD development typically starts in midlife, and metabolic disturbances during this period, such as obesity, are associated with increased AD risk [106, 107]. We and others have shown that obesity exacerbates AD-like pathology [108–110]. For example, we recently showed that feeding TgF344-AD rats the HFHS diet during the early prodromal period negatively impacts AD trajectory [53]. Collectively, that evidence suggests that obesity and associated comorbid conditions during early AD increase AD risk. We speculate that obesity is a risk factor for AD, at least in part, because it is an early symptom of AD.

There are limitations to the present research that should be addressed in future research. For example, our analysis of AD neuropathology was limited to soluble forms of Aβ, and future studies should examine whether the HFHS diet promotes the production and deposition of ptau. Although the correlations we observed between hypothalamic Aβ_42_ and peripheral metabolic dysfunction suggest that increases in hypothalamic Aβ_42_ induce disturbances in BAT and glucose regulation, future work should manipulate hypothalamic Aβ_42_ to assess causality. The present study does not reveal the causes of the sex-dependent increases in body temperature that were observed in male and female TgF344-AD rats. It would be interesting, for instance, to determine whether fasting rats during the onset of the dark cycle prevents the temperature increases seen in the dark in female TgF344-AD rats, which would be consistent with our hypothesis that the increased body temperature is due to heightened intake. Further, future studies could employ indirect calorimetry, which is the gold standard for assessing energy expenditure [111, 112], to determine whether increases in body temperature are associated with increased energy expenditure or conversely whether the increases in body mass and decreases in lean-to-fat ratios are linked to decreased expenditure. Finally, we note that whether the male or female TgF344-AD rats weigh more than WT rats seems to vary within and across laboratories [37, 41, 45, 51, 82, 113–116]. For instance, our findings show that TgF344-AD males who are 4–7 months old weigh more than WT rats (Fig. 1d) and yet in another cohort we do not observe any differences in body mass at a similar age (Fig. 3c). Even when male TgF344-AD rats do not weigh more than WT rats, they do have lower lean-to-fat ratio (see Fig. 3c vs. 3f), which is more predictive of health than lean or fat or body mass alone [117–119].

In summary, we used a multisystem approach to provide a comprehensive analysis of peripheral energy balance in the context of early hypothalamic amyloid accumulation. We show that energy homeostasis is disrupted in TgF344-AD rats during early AD development in a sex-dependent manner and that some of these changes are associated with hypothalamic Aβ_42_ levels. The presence of sex-dependent associations between hypothalamic but not cortical Aβ_42_ levels and measures of disrupted energy homeostasis highlights the need for future research into hypothalamic dysfunction in AD with mixed-sex study designs and raises the possibility that the occurrence of early disruptions of energy homeostasis may serve as biomarkers of AD or increased AD risk. By reframing metabolic dysfunction as a potential *early, sex*-*influenced consequence* of amyloid-driven hypothalamic pathology, we provide mechanistic leads with direct relevance to targeted metabolic interventions in neurodegenerative disease.

## Supplementary Information

Below is the link to the electronic supplementary material.

ESM 1 Supplementary Material 1 (PNG 284 KB)

High Resolution Image (TIF 257 KB)

ESM 2 Supplementary Material 2 (PNG 200 KB)

High Resolution Image (TIF 163 KB)

ESM3 (DOCX 20.4 KB)

ESM4 (DOCX 29.3 KB)

ESM5 (DOCX 25.7 KB)

## Data Availability

Data supporting the findings can be found online at Mendeley’s Data Repository with the 10.17632/rthpp2ktb7.1
