# Supplementary figures and images for: Hypothalamus Amyloid Levels Are Associated with Early Sex-Dependent Alterations in Peripheral Energy Homeostasis in TgF344-AD Rats
Source: Mol Neurobiol. 2026 Jul 2;63(1):739. doi: 10.1007/s12035-026-06014-4 (PMC13328149; doi:10.1007/s12035-026-06014-4)

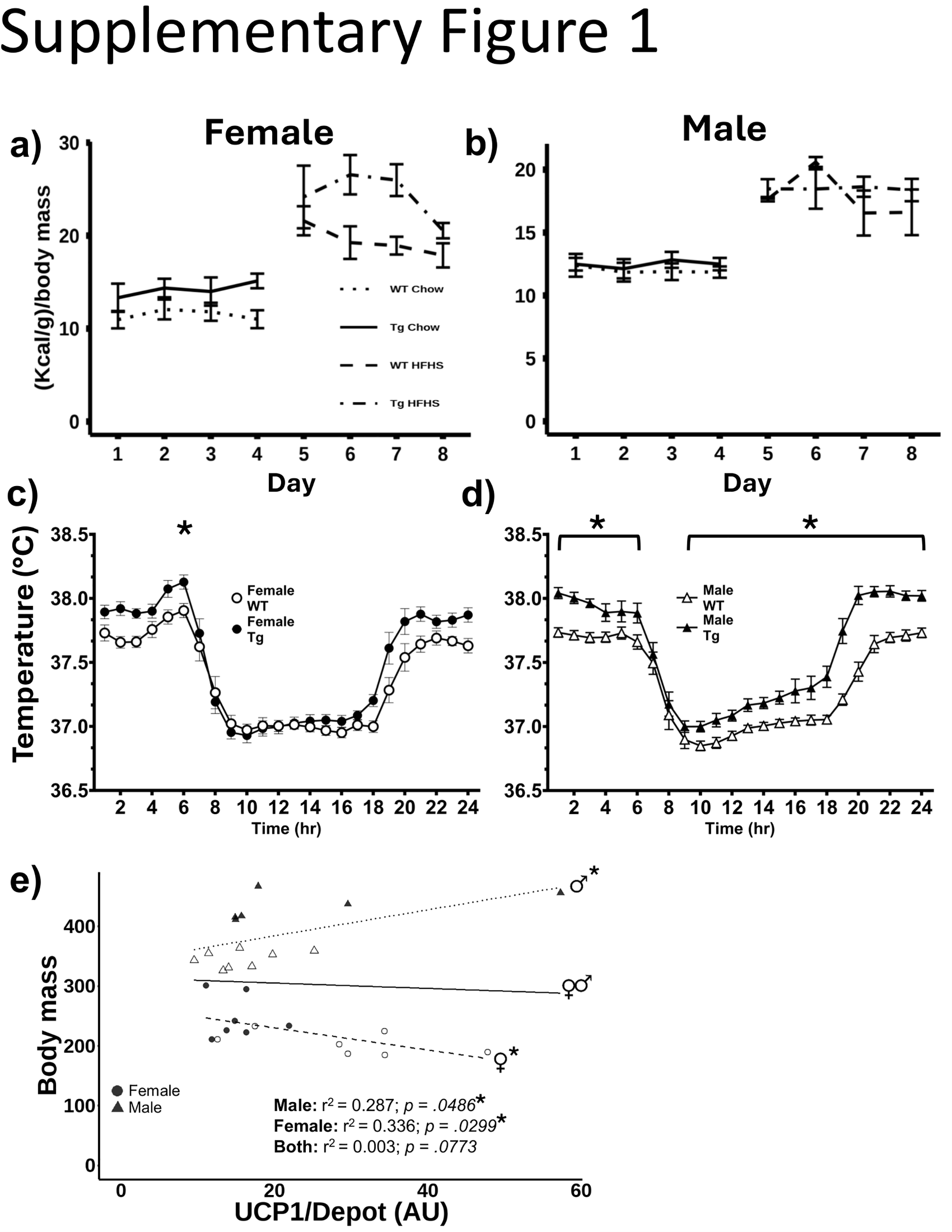

Supplement: Supplementary file 1 — Supplementary Material 1 (PNG 284 KB) [file 12035_2026_6014_Fig7_ESM.png]

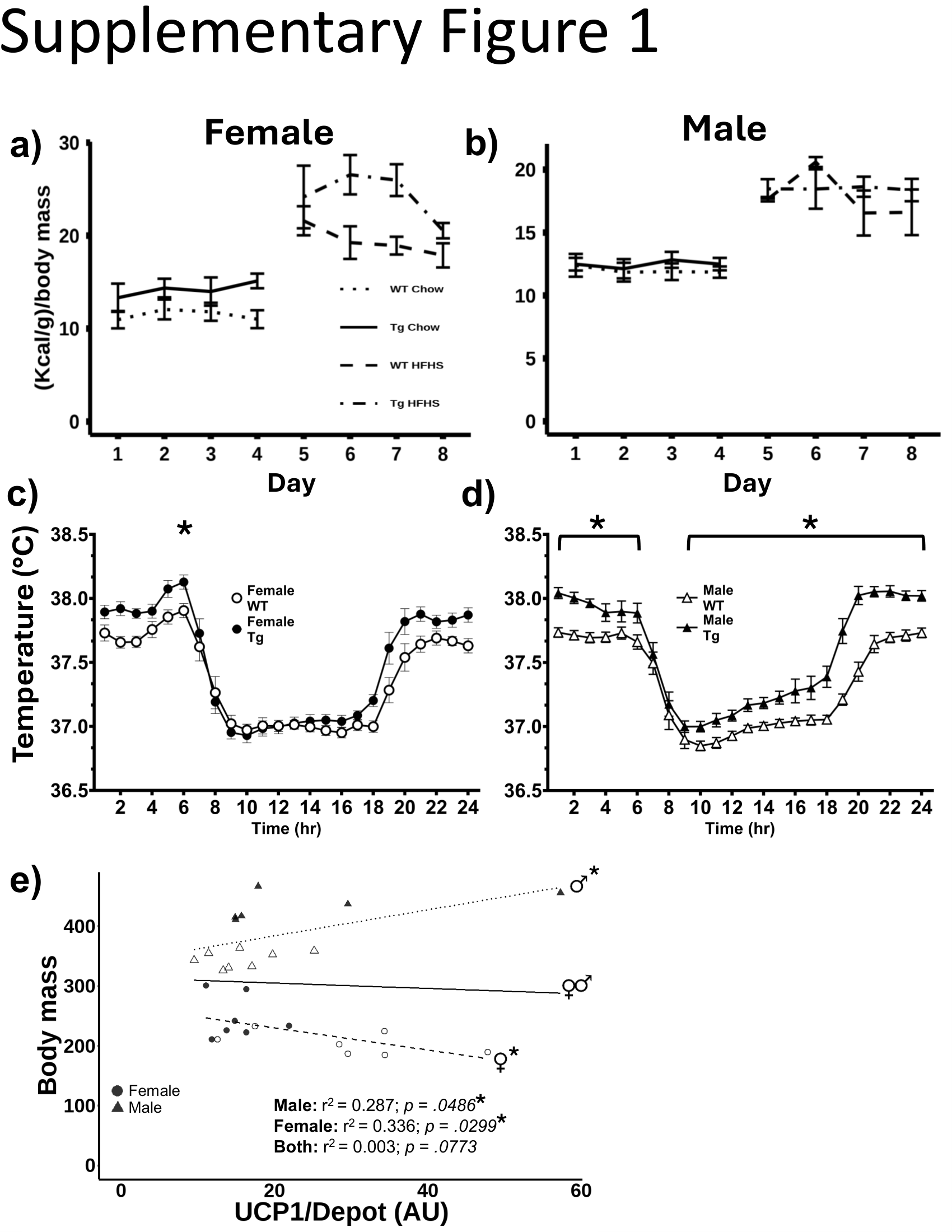

Supplement: Supplementary file 2 — High Resolution Image (TIF 257 KB) [file 12035_2026_6014_MOESM1_ESM.tiff]

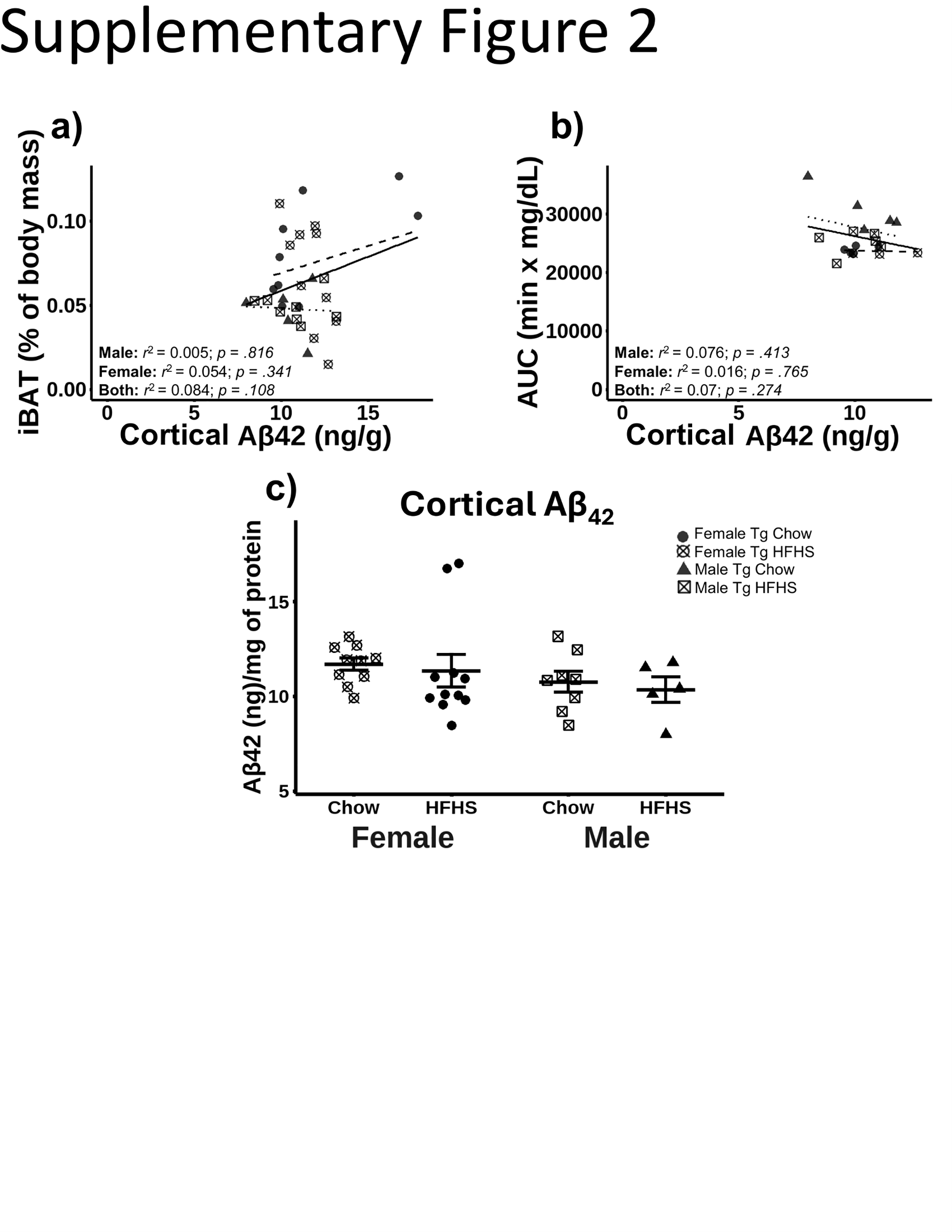

Supplement: Supplementary file 3 — Supplementary Material 2 (PNG 200 KB) [file 12035_2026_6014_Fig8_ESM.png]

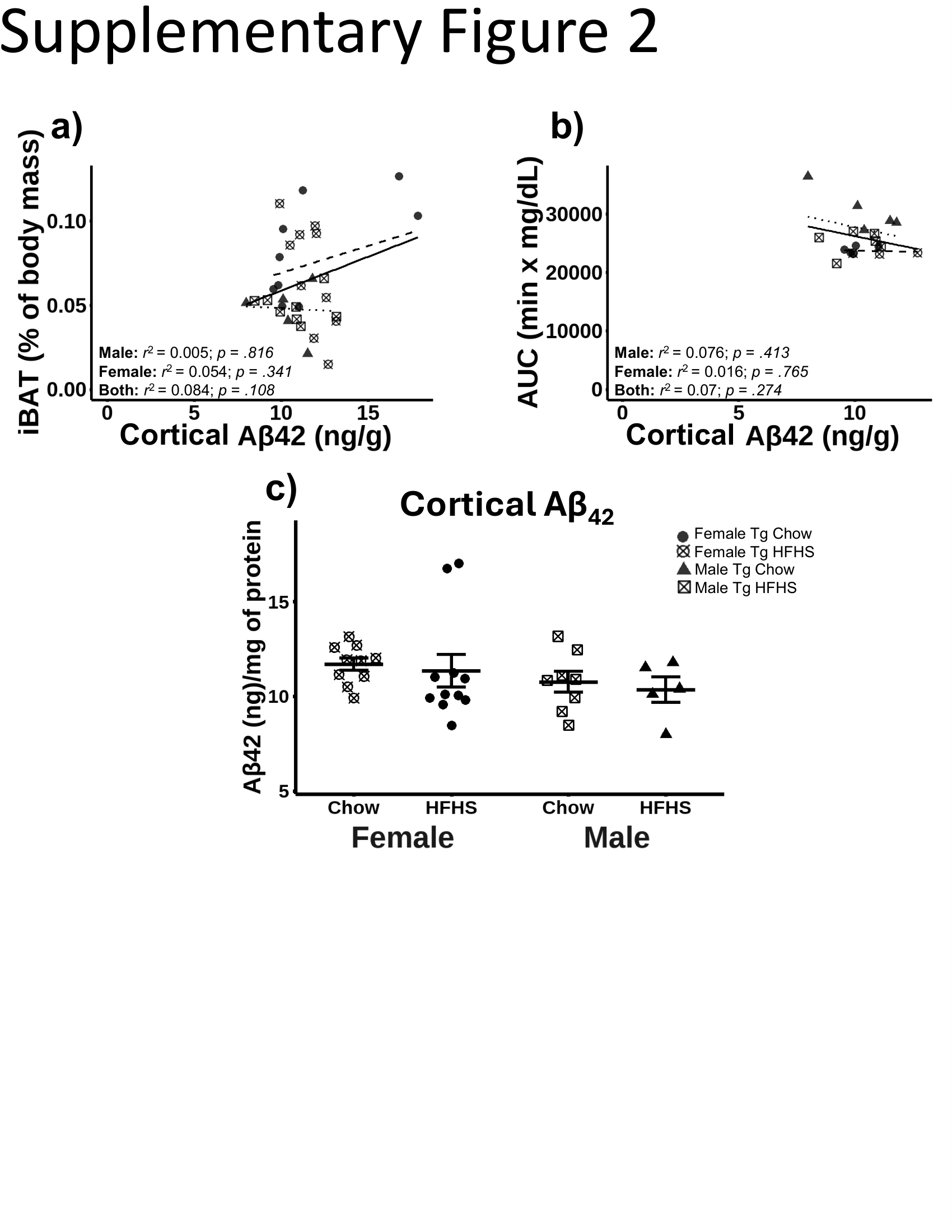

Supplement: Supplementary file 4 — High Resolution Image (TIF 163 KB) [file 12035_2026_6014_MOESM2_ESM.tiff]
