# Supplementary material for: Hypothalamus Amyloid Levels Are Associated with Early Sex-Dependent Alterations in Peripheral Energy Homeostasis in TgF344-AD Rats
Source: Mol Neurobiol. 2026 Jul 2;63(1):739. doi: 10.1007/s12035-026-06014-4 (PMC13328149; doi:10.1007/s12035-026-06014-4)
Supplement: Supplementary file 5 — (DOCX 20.4 KB) [file 12035_2026_6014_MOESM3_ESM.docx]

**S. Table 1: Caloric content of the standard chow diet and HFHS diet.​**

| **Macronutrients (% kcal)​** | **Lab Diet 5001​** | **Research Diets D12451 (HFHS)​** |
| --- | --- | --- |
| **Protein​** | **28.9​** | **20​** |
| **Fat​** | **13.6​** | **45​** |
| **Carbohydrate​** | **57.5​** | **35​** |
| **Sucrose​** | **3.25​** | **17​** |
| **kcal/g​** | **3.36​** | **4.73​** |
