# Supplementary material for: Hypothalamus Amyloid Levels Are Associated with Early Sex-Dependent Alterations in Peripheral Energy Homeostasis in TgF344-AD Rats
Source: Mol Neurobiol. 2026 Jul 2;63(1):739. doi: 10.1007/s12035-026-06014-4 (PMC13328149; doi:10.1007/s12035-026-06014-4)
Supplement: Supplementary file 6 — (DOCX 29.3 KB) [file 12035_2026_6014_MOESM4_ESM.docx]

**Supplementary Table 2: Number of rats analyzed for each measure and reasons for any exclusions.​**

|  | **Experimental outliers** | | | **Statistical outliers** | | | | | | | |
| --- | --- | --- | --- | --- | --- | --- | --- | --- | --- | --- | --- |
|  |  |  |  | **Female** | | | | **Male** | | | |
|  | **Total Tested** | **Exclusion reason** | **Total Remaining** | **WT-**  **Chow** | **WT-HFHS** | **Tg-Chow** | **Tg-HFHS** | **WT-Chow** | **WT-HFHS** | **Tg-Chow** | **Tg-HFHS** |
|  |  |  |  | **Experiment 1** |  |  |  |  |  |  |  |
| **Body mass- cross sectional** | **75** |  | 75 | 1 | NA | 0 | NA | 0 | NA | 0 | NA |
| **Early body mass- longitudinal** | **127** |  | 127 | 8 | NA | 3 | NA | 2 | NA | 3 | NA |
| **Caloric intake- food consumption** | **31** | **food hopper error** | 29 | 0 | 0 | 0 | 0 | 2 | 2 | 0 | 0 |
|  |  |  |  | **Experiment 2** |  |  |  |  |  |  |  |
| **Body temperature** | **56** |  | 56 | 0 | 0 | 0 | 1 | 1 | 0 | 0 | 0 |
| **iBAT mass- body mass** | **31** | **Collection error** | 30 | 0 | 0 | 0 | 0 | 0 | 0 | 0 | 1 |
| **iBAT- UCP1 per depot** | **30** | **Collection error** | 29 | 0 | 0 | 0 | 0 | 0 | 0 | 0 | 0 |
|  |  |  |  | **Experiment 3** |  |  |  |  |  |  |  |
| **Body mass- longitudinal** | **65** |  | 65 | 0 | 0 | 0 | 1 | 0 | 0 | 1 | 0 |
| **Body mass- terminal** | **65** |  | 65 | 0 | 0 | 1 | 1 | 0 | 0 | 0 | 0 |
| **Body length** | **65** | **Collection error** | 45 | 0 | 0 | 0 | 0 | 0 | 0 | 1 | 0 |
| **Blood glucose - GTT** | **49** |  | 49 | 1 | 0 | 2 | 2 | 2 | 1 | 1 | 0 |
| **GTT AUC** | **49** |  | 49 | 1 | 0 | 3 | 2 | 2 | 1 | 1 | 0 |
| **Blood glucose - fasting** | **65** |  | 49 | 0 | 0 | 0 | 0 | 1 | 0 | 0 | 0 |
| **EchoMRI- Fat mass** | **65** |  | 65 | 0 | 0 | 0 | 0 | 0 | 0 | 0 | 0 |
| **EchoMRI- Lean mass** | **65** |  | 65 | 0 | 0 | 0 | 0 | 0 | 0 | 0 | 0 |
| **igWAT- mass** | **65** |  | 65 | 0 | 0 | 0 | 0 | 0 | 0 | 1 | 0 |
| **rpWAT- mass** | **65** |  | 65 | 0 | 0 | 2 | 2 | 0 | 0 | 0 | 0 |
| **gWAT- mass** | **65** |  | 65 | 0 | 0 | 0 | 0 | 0 | 0 | 0 | 0 |
| **iBAT- mass** | **65** | **Collection error** | 63 | 0 | 0 | 0 | 1 | 0 | 0 | 0 | 0 |
| **Adrenal glands- mass** | **65** |  | 65 | 0 | 0 | 0 | 0 | 1 | 0 | 0 | 0 |
| **Heart- mass** | **65** | **Collection error** | 64 | 1 | 0 | 0 | 0 | 0 | 1 | 1 | 0 |
| **Liver- mass** | **65** |  | 65 | 0 | 0 | 0 | 0 | 0 | 0 | 0 | 0 |
| **Kidney- mass** | **65** |  | 65 | 0 | 1 | 0 | 0 | 0 | 0 | 0 | 0 |
| **Hypothalamic Aβ_40_** | **32** | **Low CV** | 28 | NA | NA | 0 | 0 | NA | NA | 0 | 0 |
| **Hypothalamic Aβ_42_** | **32** | **Undetectable** | 21 | NA | NA | 0 | 0 | NA | NA | 0 | 0 |
| **Cortical Aβ_42_** | **36** | **Undetectable** | 35 | NA | NA | 1 | 0 | NA | NA | 0 | 0 |
