# Supplementary material for: Hypothalamus Amyloid Levels Are Associated with Early Sex-Dependent Alterations in Peripheral Energy Homeostasis in TgF344-AD Rats
Source: Mol Neurobiol. 2026 Jul 2;63(1):739. doi: 10.1007/s12035-026-06014-4 (PMC13328149; doi:10.1007/s12035-026-06014-4)
Supplement: Supplementary file 7 — (DOCX 25.7 KB) [file 12035_2026_6014_MOESM5_ESM.docx]

**Supplementary Table 3: Morphometric measures and iBAT protein quantification​**

|  | **Female** | | | | **Male** | | | |  |
| --- | --- | --- | --- | --- | --- | --- | --- | --- | --- |
|  | **Chow** | | **HFHS** | | **Chow** | | **HFHS** | |  |
|  | **WT** | **Tg** | **WT** | **Tg** | **WT** | **Tg** | **WT** | **Tg** | **Significant**  **effects** |
|  |  |  |  | **Morphometrics** | |  |  |  |  |
| **Litter size** | 10.4 ±  0.81 | 9.18 ±  0.69 | 10 ±  1.22 | 9 ±  0.56 | 8 ±  0.82 | 8.25 ±  0.98 | 7.57 ±  1.21 | 7.38 ±  1.12 | a |
| **Body length (cm)** | 19.75 ± 0.61 | 20.33 ± 0.22 | 20.54 ± 0.19 | 20.93 ± 0.47 | 24.24 ± 0.35 | 24.58 ± 0.16 | 24.62 ± 0.67 | 24.48 ± 0.29 | a |
| **Fat mass (% body mass)** | 12.91 ±  0.4 | 15.05 ± 0.76 | 23.24 ± 1.55 | 22.87 ± 0.92 | 14.75 ± 0.46 | 15.97 ±  0.5 | 26.59 ± 1.07 | 25.88 ± 0.79 | b |
| **Lean mass** | 73.41 ± 0.37 | 72.23 ± 0.58 | 67.86 ± 2.37 | 66.32 ± 0.68 | 72.44 ± 1.15 | 71.08 ± 0.46 | 63.27 ± 0.83 | 63.97 ± 0.59 | c,d |
| **Heart** | 0.3209 ± 0.0064 | 0.2977 ± 0.0078 | 0.3011 ± 0.0115 | 0.2975 ± 0.0113 | 0.2655 ± 0.0066 | 0.2685 ± 0.0124 | 0.244 ± 0.006 | 0.2443 ± 0.0074 | a |
| **Kidney** | 0.664 ± 0.017 | 0.654 ± 0.017 | 0.583 ± 0.025 | 0.592 ± 0.019 | 0.624 ± 0.013 | 0.605 ± 0.011 | 0.546 ± 0.01 | 0.546 ± 0.005 | e |
| **Liver** | 3.067 ± 0.064 | 3.201 ± 0.104 | 2.637 ± 0.216 | 2.933 ± 0.086 | 3.031 ± 0.172 | 3.121 ± 0.119 | 3.202 ± 0.044 | 3.096 ± 0.063 | b |
|  |  |  |  | **iBAT** | |  |  |  |  |
| **Total protein (mg)** | 15.55 ± 1.84 | 14.01 ± 1.81 | 24.16 ± 4.18 | 14.45 ±  1.2 | 14.45 ± 1.56 | 8.18 ±  0.96 | 13.07 ±  1.2 | 10.16 ± 1.44 | g |
| **Protein density (µg/mg)** | 55.49 ± 3.01 | 50.98 ± 4.47 | 47.5 ±  4.62 | 38.37 ± 5.86 | 44.91 ± 5.18 | 28.75 ±  0.9 | 32.23 ± 2.69 | 32.85 ± 8.04 | g |
| **UCP1 expression (AU)** | 0.002116 ± 0.000264 | 0.001476 ± 0.000102 | 0.001154 ±  3.1e-05 | 0.000799 ±  7.6e-05 | 0.001423 ± 0.000151 | 0.001567 ±  6.5e-05 | 0.000939 ±  5.3e-05 | 0.001881 ± 0.000337 | g,h,i |
| **UCP1 (µg)** | 2.31 ± 0.26 | 1.19 ± 0.19 | 1.07 ± 0.54 | 1 ± 0.18 | 1.14 ± 0.15 | 2.32 ± 0.58 | 1.29 ± 0.42 | 3.77 ± 1.51 | g,j |
| **iBAT mass (g)** | 0.28 ± 0.03 | 0.27 ± 0.02 | 0.5 ± 0.05 | 0.38 ± 0.03 | 0.34 ± 0.05 | 0.29 ± 0.04 | 0.41 ± 0.01 | 0.33 ± 0.05 | g |
| **Footnotes** | **^a: Significant main effect of sex (p < .05); b: Significant main effect of Diet regardless of sex (p < .05); c: Significant interaction between sex and diet (p < .05);^**  **^d) Significant interaction between Diet and Genotype in males (p < .05); e) Significant effect of HFHS diet in females (p < .05) g: Significant main effect of Diet in females (^*^p^* ^< .05);^**  **^h: Significant main effect of Genotype in females (^*^p^* ^< .05); i: Significant interaction between Diet and genotype in males (^*^p^* ^< .05); j) Significant effect of HFHS diet in males (^*^p^* ^< .05)^**  **^Male: WT-Chow: n = 4-9, Tg-Chow: n = 4-9, WT-HFHS: n = 4-8, Tg-HFHS: n = 3-8; Female: WT-Chow: n = 4-5, Tg-Chow: n = 4-11, WT-HFHS: n = 3-5, Tg-HFHS: n = 3-12^** | | | | | | | | |
